# Periscope

**Published:** 1832-01-01

**Authors:** 


### Periscope;

OR,

### CIRCUMSPECTIVE REVIEW.

" Ore trahit quodcunque potest, atque addit acervo."

### I.

### On Phlegmonous Tumours in the Right Iliac Fossa. By Mr. J. M. Ferrall, Surgeon to the Maison de Santé, &c.

Our readers are aware that we have taken due notice, in different parts of this journal, of the above subject, especially as treated of by Messrs. Dupuytren, Dance, and others. It appears that M. Ferrall had collected materials for a long paper on this complaint, before the memoirs of the French authors appeared; but now considers the publication of them unnecessary. Subsequent experience, however, has thrown some light on phenomena before involved in obscurity—and having seen the complaint occasionally take a course somewhat different from that generally described, and having reason to believe that it has its origin in other causes than those to which it has been ascribed, he has selected those cases in his possession which appear to illustrate a portion of the history of the disease, of which no evidence has hitherto been adduced.

It is the chronic form of this affection which is most likely to be overlooked in practice. The attendant sickness is so like that occasioned by a capricious or dyspeptic stomach—the pain so resembles that from flatulence—that days and weeks of uneasiness are allowed to elapse before efficient steps are taken.

"Pain, more or less acute, referred to the right iliac region, is the symptom which generally urges the patient to seek advice. This leads to a careful examination of the parts; and if we find there a deep-seated immoveable tumour, extremely painful to the touch, we have the leading character of the complaint. The history of the case, with the position and feelings of the patient, and the state of the limb, will assist us to distinguish it from the commencement of abscess in the psoas or iliacus muscles, or in the abdominal parietes. On this it is necessary to dwell more particularly when describing the cases to which they bear the strongest resemblance.

When the evidence opposes this view, and we refer the phenomena to the cœcum, we have still to consider how far the tumour may be constituted by impacted fecal matters alone,—how much of its bulk may consist of phlegmonous deposits external to the gut;—and whether the neighbouring peritoneum be involved to any material extent. The condition of the lining membrane of the cœcum should also be considered, for it will appear that the tumour may in some instances be occasioned by inflammation of that membrane transmitted to the contiguous tissues, while in others it seems to arise from perforation of the intestine by ulceration, and the contact of its fecal contents with the cellular structure immediately on its outside."

On examining tumours in the iliac region, we sometimes meet with cases which do not arrange themselves under the class of merely phlegmonous tumours, in the general acceptation—for it will sometimes be found that the tumour consists of scybalous masses distending the cæcum, and occasioning colicky pains and other phenomena attendant on phlegmon in the same situation. Even flatulent distention of the cæcum is often distressing. A sense of tension here is not unfrequently felt on taking horse-exercise soon after meals. The pain is acute—is relieved by pressure—and generally goes off in a few minutes. The distention produced by feculent matter is a more serious, because less transient, source of uneasiness, and pressure here gives pain. "A full colon has occasionally been treated for hepatic disease; and the diagnosis been apparently justified by tenderness on pressure, and resistance and tension in the muscles which protect the hypochondriac and epigastric regions."

That vascular congestion obtains in most cases of painful tumour of the cæcum, the author is ready to admit; but that we may have a painfully distended cæcum, without any marked evidence of inflammation, he is satisfied. M. Meniere, however, is of a different opinion, and believes the tumour to consist, in every instance, of deposits external to the intestine, considering the inflammation as the primary and essential condition of the complaint. He looks on the constipation as altogether accidental, neither constituting the bulk of the tumour, nor having any share in the production of the disease. Mr. F. has come to a different conclusion, and offers cases to support his opinions. The following one is sufficiently convincing.

"*4th July*, 1823.—I was requested by M. K. to see Margaret Hopkins, aged 18, the daughter of his housekeeper. She had passed a sleepless night, raving constantly. She was tossing about in the bed, and kept her eyelids forcibly closed; talked incoherently, but when questioned complained of pain in the right side of the abdomen. On examination, (to which she was very averse,) the right iliac region was found to be swelled, hard, and apparently very tender. The remainder of the abdomen was pliable to the touch. Bowels confined for the last five days, but she was yesterday apparently in good health; tongue loaded at the back part, clear white anteriorly, and along the sides; skin hot; pulse when first felt (which was immediately after the examination of the tumour) 120, and small. This suggested the idea of inflammation; but before I left her I found it reduced successively to 100, 90, and finally to 75. She was thirsty, but there was no irritability of stomach.

The abdomen ordered to be fomented, and an enema to be thrown up, (containing *Oleum Terebinth.* and fœtid tincture,) every fourth hour *ad effectum.*

Vespere.—She is better. An hour after the enema was administered a quantity of indurated fæces came away. The enema was repeated with continued effects. She is now free from pain and perfectly composed; skin moist; pulse 70; no trace of swelling or tenderness is discoverable in any part of the abdomen."

No doubt a prolongation of the impaction of the fæces would have led to inflammation in this case—but we think there is no evidence of its having commenced when the bowels were cleared.

*Case* 2. 13th August, evening. Mr. L. was seized with pains in his bowels, accompanied by nausea and general illness. Various purgatives were tried, but they all returned. On the 18th our author saw the patient. The abdomen was full, but yielded readily to pressure till the hand traversed the right iliac region, where there was a good deal of tension and tenderness to the touch. This fulness was found to occupy but a limited space between the spine of the ileum and the middle line of the abdomen. The tumour was deeply seated, but the parietes rolled freely over it in certain positions. His tongue was thickly coated—pulse 90—skin hot and dry—lay on his back, with his knees drawn up. Enemata and the hip-bath. 19th. He was better, some hard scybala having come away. The abdomen was less full; but the iliac tumour was perceptible to the eye. Enemata, leeches, hip-bath—a grain and a half of calomel every four hours. 21st. Mr. Colles, of Dublin, visited the patient with Mr. F. Some more scybala had passed—and the bulk of the tumour was diminished. Calomel continued—enemata every six hours. 22d. Motions now fluid, and nearly of a natural colour — abdomen reduced—swelling much less. 24th. The mercury has affected the breath and the gums—dejections fluid and yellow—the swelling in the iliac region nearly gone. Calomel discontinued. From this period he went on well, and gradually recovered health.

"Mercury does not appear to enter into the treatment at the Hôtel Dieu; but while we are engaged in lessening the inflammation by depletion, we should not lose sight of the propriety of attempting the removal of the effects already produced by diseased action, as far as it has gone. Independent of the coagulable lymph thrown out in the cellular tissue surrounding the *cæcum*, it is probable that some degree of thickening has taken place in the mucous and peritoneal coats. Though this may subside in the progress to convalescence, it may, on the other hand, remain and become the nucleus for disease at a more distant period, when a resumption of imprudent habits shall have exposed the parts to fresh irritation. Chronic disease in this situation is not very uncommon; and with this experience it becomes our duty to institute, as early as possible, a plan for promoting the absorption of effused fluids, and the restoration of healthy action in the parts. The peculiar power of mercury is here favourable to our purpose, and after the reduction of the urgent inflammatory symptoms, generally fulfils the indication with tolerable certainty. A case is lately detailed by the Editor of the Medico-Chirurgical Review, in which this remedy was employed by himself with the best effects; and, as far as I have observed, I believe it will be found to constitute a very efficient instrument in combating this dangerous affection."

Some cases are detailed by Mr. F. where inflammation and suppuration of the cæcum took place, but these we shall not notice at present. We point the attention of our readers to the more common and more remediable affection—impacted fæces—an affection too often neglected because not suspected.—*Ed. Journal.*

---

### II.

### Dothinenterite. By Dr. Corrigan.

We have, on several occasions, differed with Dr. Corrigan on physiological points—we are happy to have an opportunity of agreeing with him in pathology. In the last number of our Edinburgh contemporary, Dr. C. has published some hospital reports, and, among others, a case of dothinenterite, (which, by the way, the Doctor persists in calling dotientherite, contrary to M. Bretanneau and its Greek derivation,) with some judicious reflections. The case was as follows.

*Case.* The patient was a young man of temperate habits, whose illness commenced, ten days before the Doctor saw him, with lassitude, and dull pain across the eyebrows, which symptoms continued with varying intensity up to the morning of the first visit, when stupor set in. The Doctor had some difficulty in rousing him. His countenance was pallid and congested—eyes dull, but not diffused—sensible to light, but not pained by it—feet and skin cool—pulse 80, labouring, slow—tongue clean—bowels confined—abdomen soft. Dr. C. took blood from the temporal artery, and ordered the head to be shaved. Calomel was administered in large doses—turpentine enemata were exhibited—and the feet fomented. Next day there was less stupor. Skin cool and covered with small petechiæ. The bowels had been freed but once—tongue clean. Venesection to twelve ounces—a senna mixture—large blister to the head—enemata continued. The pulse, immediately after bleeding, rose to 100, and became free. In the night, delirium took place—and on the next day, (13th of his illness,) there was intolerance of light and noise—twitchings of the muscles—pulse 120—hot skin—brown tongue—tender abdomen—free bowels. Calomel, with ipecacuanha, in small doses, was given every two hours. Next day, the delirium continued—the pulse rose to 134, full and vibrating—stools and urine became involuntary—abdomen tender and shrunk. He died rather suddenly on the next day but one, the sixteenth of the fever, after a quiet sleep of five hours.

*Dissection.* "The vessels of the dura mater and pia mater were very turgid, and all the large venous trunks were distended with colourless coagulum. The substance of the brain was very vascular, and the ventricles contained four or five ounces of clear fluid. The mucous membrane of the stomach and intestines (speaking generally) was natural in colour and consistence. The mucous glands of the duodenum were enlarged and hardened, so as to give to the finger, when passed over them, a sensation as if small grains of shot were imbedded under the mucous coat. Small ulcerations in great numbers were spread along the internal antimesenteric surface of the jejunum and ileum, and about the *caput coli.* The edges of the ulcers were hard; the mucous membrane around them not inflamed. These ulcers seemed to be the last stage of the disease of the glands, the commencement of which was the enlargement and hardening described as existing in the duodenum."

Dr. C. very naturally asks, was the death of this patient owing to the ulcerations in the intestines, a conclusion to which the disciples of Bretonneau, Louis, and even Broussais would come at once? After much close and appropriate reasoning, Dr. C. arrives at a different conclusion, namely, that there was quite enough of disease in the head to occasion death, and to that organ the symptoms, during the disease, were unequivocally referrible. The abdomen was not complained of till the 11th day of the illness, and the bowels up to that time were confined.

"Weighing all these considerations, we cannot but come to the conviction, that the French opinions are not well founded, and that our own practice of viewing symptoms in fever, (generally speaking,) as direct signs of diseased actions in the organs, whose altered functions furnish them, is better calculated to lead us to an accurate diagnosis and a safe treatment. If we compare the results of treatment, we find little encouragement to exchange our views for those of the French. In our treatment the mortality is from one in eight to one in twelve. From Louis's own account, the mortality in treatment directed by the opinion, that the fever in question has its seat in the Peyerian glands, is one in three."

Some of the Broussaians would be wicked enough to say or insinuate that the "large doses of calomel" and the turpentine enemata, exhibited at a time when the brain was oppressed and the intestines consequently in a torpid state, were the chief causes of the ulcerations in the bowels. Certain it is that the abdomen became tender on the second day after these means were used, though we do not aver that this tenderness was the consequence of the remedies. Nevertheless we think we have seen mischief done by heroic medicines given where the brain is oppressed, and where the intestines will not respond to medicines in the same manner as when the brain is not labouring. The grand object is to relieve the organ which is most affected, and that, if possible, by means applied near the organ itself. By this procedure we obtain our object, if attainable, without paying too high a price in the injury of another part. This is a practical hint worth bearing in mind.

---

III.

### MOLLESCENCE OF THE BRAIN—SEROUS APOPLEXY—NERVOUS APOPLEXY.

[Hôtel Dieu.]

I. MUCH discussion has taken place respecting the nature and cause of softening of the brain. The earlier pathologists, when they met with this condition of the organ, did not attempt to account for it—and, under the name of apoplexy were ranged the various alterations of the cerebral mass. Valsalva and Morgagni regarded mollescence of the brain as a kind of decomposition or putrefaction, the effect of which was suddenly fatal. When their scalpel detected this state of the organ, they looked upon it, not as the cause of the disease under which the patient had laboured, but as the final termination of the malady. Bayle, Cayol, Recamier, and other modern pathologists, have drawn attention to the subject under consideration—carefully describing the appearances, and cautiously endeavouring to account for them. Recamier designates the morbific cause by the old name "ATAXIC FEVER"—and the effect he terms "foyer ataxique." Rostan is more cautious. He minutely details the symptoms and post-mortem appearances, styling the affection an "organic alteration," which, of course, prejudges nothing as to the nature or cause. Lallemand indeed is more bold. He ascribes the ramollissement at once to an inflammatory process—and in the majority of cases, there can be little doubt that this condition of the brain is the effect, the termination of encephalitis. On the other hand, when in and around the softened portion of brain, we can discover no sanguineous injection, no purulent infiltration, no morbid secretion whatever—nothing but a mere diminution of consistence in the nervous pulp—how can we call this inflammation? Andral doubts the correctness of the exclusively inflammatory doctrine —and in a report from the HÔTEL DIEU, published in a recent number of the JOURNAL COMPLEMENTAIRE, there are some cases related to shew that Lallemand's hypothesis is not quite correct. Some of these we shall here notice.

*Case* 1. A female, aged 68 years, entered the HÔTEL DIEU, on the 14th June, 1830, for a slight bronchial affection, for which she was treated in the usual manner, and soon recovered. On the 1st of July, this woman, while walking about the wards, suddenly fell down deprived of sense and motion. The pupils were contracted, the face pale, the respiration quick, the pulse hard and frequent. The case was pronounced, of course, to be apoplexy—and venesection, sinapisms, &c. were ordered. She lingered in a wretched state for 25 days, when mortification of the integuments of the back put an end to her existence.

On dissection, there was no turgescence of the vessels of the encephalic membranes. These membranes were rather pale than otherwise. The anterior portions of each hemisphere appeared natural; but on approaching the middle lobes, they were found without consistence, and a certain portion reduced to a kind of bouillie, where all trace of cerebral texture was lost. There was no mark of inflammation, or even injection in any part of the brain.

*Case* 2. This occurred in the hospital for old women. A female, aged 79 years, of apparently good constitution, had been in the Salpètrière since the 13th July, 1830. She was carried to the above institution on the 25th April, 1831. She had complained, for a fortnight, of her head in the right side, while there was numbness or sense of formication in the extremities of the opposite side. There was some impediment in her speech. She ate and slept well. The left arm gradually lost its motile power, and the lower extremity was benumbed. The other side possessed motility and sensibility.—There was little disturbance in the other functions. For a fortnight, or so, the paralysis gained ground, and then she remained hemiplegiac for a month or more. Pains then came on in the affected parts—agitation and delirium at night succeeded—the evacuations became involuntary—and death closed the scene.

On dissection, the meninges of the brain appeared gorged with fluid blood, which also flowed freely from the sinuses of the dura mater. There was nothing unusual in the external character of the cerebral mass ; but, on penetrating some way into the right hemisphere, a focus of mollescence (foyer d'un ramollissement) was discovered, six inches in length and two in breadth. There was no other organic alteration of any consequence in the brain.

II. *Serous and Nervous Apoplexy.* Is there such a disease as serous apoplexy? When, on opening the body of a patient who has died with the usual symptoms of apoplexy, we find a considerable effusion of serum either between the meninges or in the ventricles, without any other phenomena to account for death, we say, with Morgagni, that, in such cases, the brain is compressed by the water, in the same way that, in sanguineous apoplexy, it is compressed by blood. Magendie, indeed, has shewn that, in health, there is a certain quantity of serous fluid, both in the ventricles of the brain, and in the spinal canal—and that, in old people, there is a diminution of the brain, and a corresponding increase of water. M. Serres has made experiments which seem to shew that the brains of animals will bear a considerable degree of pressure with impunity. Finally, we find effusion to a considerable amount, after death, in cases where there were no symptoms of apoplexy before that event. But we must not be led astray by anomalies, which abound in physiology and pathology. Nothing can be more certain than that a preternatural collection of fluid in the ventricles or between the meninges is dangerous or fatal, according to the degree of accumulation. In the great majority of these serous effusions there is previous inflammation or vascular excitement—but it must be admitted that in some cases, there are proofs of such inflammatory origin; and hence we have reason to conclude that a morbid collection of serum may suddenly take place independent of congestion, or at least of inflammation. We shall glance at one or two cases brought forward, in the HOSPITAL REPORT already alluded to, in the JOURNAL COMPLEMENTAIRE.

*Case* 1. (HÔTEL DIEU—Clinique of M. Rostan.)—A female aged 87 years, of good constitution, and previously enjoying good health; became suddenly insensible, on the 23d of February, at 10 o'clock in the morning. Hemiplegia of the right side was complete. Examined immediately, there was observed some disturbance of the circulation, and some spasmodic movements, and a natural ptyalism. The paralysis appeared to commence in the lower extremity, then affected the upper, and lastly the tongue. There were clearly defined intervals between these phenomena, apparently indicating the successive stages of accumulation in the head and spine. On the 24th there was profound coma, with febrile heat, and general re-action. 25th. The re-action more decided—the patient was able to give some account of her sensations and preceding ailments. She stated that she had had pain in her head, with giddiness, and occasional loss of sense for a year past. Sixteen leeches were applied to her neck, and laxatives were given. For some days after this, she appeared to get better. Her speech was improved — motion and sensation were in some degree, restored to the right lower extremity. In the beginning of March, however, her strength declined — all re-action ceased — the tongue became dry. Tonics were tried —but she died on the 10th of March.

The meninges were in their natural state. So did the brain itself appear. On making incisions into its substance, no injection was visible. The parietes of the ventricles, however, were pointed with blood, and the inner surface dark-coloured with the same. The ventricles themselves were greatly distended with a pellucid fluid, amounting to at least six ounces. The third and fourth ventricles were filled with the same kind of fluid. There was no other morbid phenomenon in the head, which was carefully examined by M. Rostan himself, who had prognosticated that a sanguineous effusion would be found on dissection.

*Case* 2. A female, aged 28 years, of apparently good constitution, but enfeebled by several accouchments and by the development of a scirrhous ovarium, was seized on the 15th March last, after some hours' vague complaints, with vertigo, a degree of general insensibility, and complete loss of speech, attended with some convulsive movements of the upper extremities. The pulse was calm and regular—the skin rather cool than otherwise—the breathing slightly accelerated—the expression of countenance much altered. A physician was called in and prescribed; but in a few hours she revived considerably, though there was still much loss of sensibility. She was able to speak tolerably clear. 24 leeches were ordered to the head, and sinapisms to the feet. It was pronounced to be a case of sanguineous effusion in the head. In the evening, there was paralysis almost complete of the right side, with some arterial re-action. The pupils were dilated—the speech much embarrassed, but not entirely extinguished. It remained in nearly the same state during the 16th—and on the 17th paracentesis abdominis was performed, and ten pints of fluid were abstracted. As the water flowed from the puncture, she appeared to regain sensibility, and when the operation was over, she was very sensibly improved. Next day (18th) she made many efforts to speak, but was only able to articulate the word *Amen.* At nine o'clock the same evening, while being transported from one bed to another, she uttered a cry indicative of great pain, and expired five hours afterwards in great agony.

*Dissection.* We need not describe the appearances in the abdomen, where a vast ovarian disease existed. There was much serous infiltration throughout the whole body, and this was observed between the meninges of the brain. In the middle part of the left hemisphere there was observed a roundish patch of sanguineous ecchymosis, an inch and a half in diameter, and a quarter of a line in depth. The sinuses and the vessels of the brain were immoderately distended with blood. The cerebral pulp appeared sound. The ventricles and the spinal canal were filled with limpid serum.

*Nervous Apoplexy.* The next question discussed by the Hospital Reporter, is the existence or non-existence of nervous apoplexy. The term nervous he defines to be an alteration in the vital powers or properties. But he confesses that he cannot conceive any such alteration except as in connexion with a morbid alteration in the organic structure. So far he denies the existence of nervous apoplexy. "But if," says he, "by nervous apoplexy we mean a sudden death without any appreciable organic change—an instantaneous stroke of the nervous system, which causes death without altering, as far as we can discover, the organization of any part, then there is such a disease." There is an apoplexy which is neither sanguineous nor serous, but something different from both, and as yet unascertained as to its true nature. The following case is reported by M. Cruveilhier, as an instance.

*Case.* M. D. a surgeon-dentist, aged 23 years, of good constitution, and in excellent health, was remarkably well on the fourth of April. He dined at six o'clock, and then went out to walk. He suddenly fell down on his face, and died instantaneously. M. Cruveilhier was at hand, but life was entirely extinct. On dissection, the integuments of the head were found injected. On removing the cranium the dura mater appeared livid—and all the sinuses and veins were gorged with black blood. The substance of the brain itself presented nothing particular. M. Cruveilhier considered all these phenomena as secondary and consecutive. The congestion of vessels were the effect or sequence, not the cause of death, which he considered as NERVOUS APOPLEXY.

This may admit of doubt. It might perhaps be equally well styled a blood-stroke (coup de sang) as a nervous affection. But we are ready to grant that the nervous system, in such a case, is the first affected, and the vascular system the second.

---

### IV.

### Cancer of the Stomach mistaken for Hypertrophy of the Heart—Perforation of its Coats, without Extravasation into the Abdomen. Observed in the Hospital St. Antoine.

*Case.* Adrian Hubert, aged 54 years, began to experience symptoms of disease three years ago, and for which he had been in several hospitals, with little relief—or rather with positive disadvantage. In one place he was treated for disease of the heart, because of frequent palpitations of that organ—in another, it was thought that there was some mechanical obstruction in the venous system, from the œdematous state of the hands and feet—in a third, the disease was pronounced *gastro-enterite*, the patient complaining much of pains in the abdomen, and diarrhœa—while, finally, there appearing symptoms of icterus, he was pronounced to have hepatitis. In the ST. ANTOINE hospital they fell into error, as did their predecessors. The patient, on his entrance (17th of April) complained only of palpitations in the cardiac region, flying pains of the chest, and some difficulty of respiration. The pulse was 100 in the minute. No pulsation of the heart was felt by the hand, but the ear heard a distinct bruit, which, however, varied much in intensity and regularity. As the medical officers of the hospital were then much employed in testing the effects of digitalis on the action of the heart, it was determined to exhibit it in this case: but there being some symptoms of gastric irritation or inflammation, these were first combated by proper means and rigorous diet. The appetite returned, but the patient still referred to pain in the region of the heart. Food was now permitted, and on the 29th April, the digitalis was commenced—*six grains of the powder daily*. This dose was continued till the 3d of May, without making much impression on the pulse, which was at 70 in the minute. In the following days the dose of digitalis was increased to nine grains, and the pulse came down to sixty. On the 6th of May the patient complained of colicky pains in the belly—diarrhœa came on in the evening—vomiting followed the last dose of the digitalis—and the countenance expressed great sufferings. The digitalis was, of course, suspended, and food, which had hitherto been allowed freely, was restricted to gum and rice-water, with a single plate of soup. The pains gradually lessened—the diarrhœa ceased—and the patient once more got some appetite for solid food; but was still kept low. On the 15th of May, the scene greatly changed. The œdema which had hitherto been confined to the feet, now suddenly extended to the whole of the legs and thighs—the prostration of strength was great—the pulse became rapid and feeble—nausea was constant—the respiration embarrassed—the epigastrium exceedingly tender and painful—the stools liquid and frequent—the tongue red at the point—the countenance yellow and sallow. He lingered out a wretched existence till the 7th of June, when he was relieved from his sufferings by the hand of death.

*Dissection.* The lungs were perfectly sound and crepitous. On opening the pericardium a patch of adherence between that and the heart, the size of a half-crown, was observed; but without any lesion of the central organ of the circulation whatever. The heart was not at all enlarged—its valves and chambers all in a natural state. The liver was black, and parts of it softened. The gall-bladder was shrunk to nothing, and perfectly void of bile. In the small curvature of the stomach was a vast ulceration, with complete perforation; but the contents of the stomach were prevented from being extravasated by a fold of the little omentum, which formed a kind of plug to the perforation—"etait introduite dans la perforation, et l'obstruait à la manière d'un veritable bouchon." There were several other ulcerations, some large and some small, with considerable traces of inflammation. Near the pylorus was situated an enormous fungoid tumour, the size of a man's fist, the neighbouring coats of the stomach being greatly thickened and indurated. The surface of this tumour was ulcerated, and through its centre ran the perforation before alluded to, and which was corked by the piece of omentum. The pyloric orifice was not affected, and consequently the chyme passed readily from the organ, which was not augmented in capacity. The cancerous mass evidently pressed upon the ascending cava, and thus, in all probability, produced the dropsical infiltrations.—REV. MED.

How this case could have been mistaken for hypertrophy of the heart, it is difficult to imagine; but it is easy enough to conceive that the enormous doses of digitalis exhibited to this unfortunate patient did not contribute much to his comfort, or to the mitigation of the dire disease that was going on in a vital organ !

---

V.

Observations on Syncope and Cerebral Congestion. By M. Piorry.

The above subject occupies the 5th section of M. Piorry's Collection of Memoirs, and deserves some notice in this Journal.

1. *Causes of Syncope.* The general opinion, corroborated by Bichat, was, that syncope was entirely owing to suspension of the heart's action. But it is to be remembered, says M. Piorry, that many of the exciting causes of syncope, as moral impressions, odours, the sight of disgusting objects, &c. can only act on the brain or the organs of sense. Is it the same in syncope from hæmorrhage ? Let us, says our author, interrogate the facts. It is rare that, in syncope, the action of the heart completely ceases. In hæmorrhage, the cerebral functions fail before the contractions of the heart. The latter organ beats a long time, though feebly, after the sensorial functions are suspended. The heart is found to beat for some time after an animal has been bled to death. If a man or animal be kept with the head elevated, after a considerable quantity of blood has been lost, syncope will take place, although the heart will be heard to beat in the chest, and even the pulse felt in the arteries of the lower extremities. " It is, therefore, in the brain that lipothymia commences." If we lower the head, and raise the inferior extremities, syncope will cease—hence we may conclude, that it is the restoration of cerebral excitement which puts an end to syncope. Those syncopes (says our author) which are produced by certain moral impressions and sensations, are evidently the result of defective action in the brain, the same as in the other cases. Pullen, indeed, separates these two kinds of syncope, placing those from hæmorrhage to the account of the heart—those from moral impressions or physical sensations to the brain ; but Bichat, with whom our author disagrees, traces both classes to the same origin—the heart—M. Piorry, to the brain, conceiving that the heart is only secondarily affected.

*Diagnosis of Syncope from Cerebral Congestion.* To discover that an organ is suffering requires not much skill ; but to ascertain the mode in which it is labouring is not quite so easy. If irritation was always the same in its nature—" if diseases were, in all cases, alterations in *plus,* and never in minus —in short, if there were no specific diseases, diagnosis would be a much easier study than it is. But reason, as well as experience, shews that there are different kinds of irritation—and it is in the endeavour to maintain its identity in all cases, that the disciples of Broussais have thrown themselves most off their guard. An organ will often exhibit the same symptoms from excess, and from defect of stimulus. Thus, when the retina is over-excited by intense light, indistinct vision is the result. The same takes place when we pass from a very clear light to an obscure one. The voice becomes hoarse when the larynx has been much exerted —and the same thing often occurs if we remain several days without exercising this organ. An instance of this is related by the author, but we imagine it is far from being a general phenomenon. The stomach becomes irritated, painful, and even inflamed, from excesses of the table :—the same phenomena occur when hunger is long sustained. The disease called hemicrania is occasioned by plethora, and also by inanition. The muscular fibres of the stomach, bladder, rectum, &c. contract by the excitation of their contents, or the influence of the nerves distributed to them. They contract also convulsively when they are deprived of their due supply of blood, or when the brain ceases to receive its supply of the vital fluid. Finally, observes our author, the phenomena of syncope and of apoplexy, or cerebral congestion, display an analogy and similitude that, he has no doubt, causes them often to be confounded. The distinction, however, is of vast importance, and M. Piorry proceeds to inquire, if there be any functional symptoms which can tend to elucidate the diagnosis.

1mo. In cerebral congestion, or apoplexy, there is suspension, more or less sudden, of the intellectual functions:—The same in syncope.

2do. In the *former* disease, there is a suspension of function in the organs of sense:—The same occurs in lypothymia. In both cases, we sometimes see spasmodic contractions, sometimes loss of power of the members.

3tio. The drawing of the mouth and the partial paralysis have been laid down as pathognomonic signs of effusion on, or some disorganization of the brain; yet our author has seen these phenomena unequivocally in syncope. The same may be said of convulsive motions in the eyes and muscles of the face—involuntary evacuations—stertorous breathing, &c. The state of the circulation furnishes more certain diagnostic marks. The contractions of the heart, in cerebral congestion, are slow, soft, and easily analyzed. In syncope, they are accelerated, weak, and very irregular. The pulse in general, and especially in the arteries about the head, is full, vibrating, and slow in apoplexy—the reverse in syncope. In the *former*, the face is red—in the *latter*, pallid. But these indications are not always so very clear in the state of the circulation. Cerebral congestions or apoplexies sometimes supervene in individuals, whose circulation is languid and pulse feeble—whose faces and lips are habitually pale. Thus, he observes, the diagnosis between syncope and apoplexy, in many cases, is very difficult; and yet it is of the greatest importance that these two affections should be accurately distinguished from each other, as they require diametrically opposite treatment. The author is quite sure that the two affections are often confounded—acknowledging that he himself has committed the mistake. Indeed he relates a case of this kind. The diagnostic mark which M. Piorry relies on is altogether a physical one. It consists in the *position* of the patient. If the phenomena depend on syncope, the horizontal position will ameliorate the symptoms, and vice versâ. If, on cerebral congestion, the vertical posture will relieve, while the horizontal will aggravate the symptoms. In the latter case (cerebral congestion) M. Piorry recommends ligatures to be placed on the extremities, to produce a stasis of the blood in those parts. We much doubt the utility of such a procedure. If ligatures prevent the return of venous blood from the limbs, it also prevents any arterial blood being sent to them. The thing, therefore, is as broad as it is long.

II. ON DEATH FROM SYNCOPE—OBSERVATIONS ON VENESECTION IN GENERAL.

The cessation of the cerebral circulation is, our author thinks, next to asphyxia from the accumulation of bronchial mucus, the most common way in which death takes place. In all great hæmorrhages, there is an abundant secretion of fluid from the surface of the intestinal canal, and also from all the other mucous tissues, which tends further to exhaust the strength and deprive the brain of a due afflux of blood. The heart being ill supplied with nervous energy from the brain, becomes distended, and at length ceases to carry on the circulation. From the circumstance that the brain is encased in bone, the vessels will be found full after death, even from hæmorrhage—and this has led some pathologists astray. Our author has often seen death occasioned by injudicious venesection. In dilatation of the heart, at the Hôtel Dieu, he has often seen the fatal effects of bloodletting. The author therefore lays down a set of rules respecting venesection, which he thinks it would be of great use to observe.

1mo. When, in a person, we find a dull sound on percussion of the chest—when there is active dilatation of the heart—when the liver is enlarged—the veins distended, the pulse full, and the circulation active, we may be sure that the circulation is in excess, and we need not fear to bleed freely. But if the symptoms are the reverse of the above, we should be cautious in the use of the lancet. We should also be cautious, he observes, when the skin is fresh-coloured, but where the internal organs give indications the reverse of plethora. In doubtful cases, we should never bleed in the horizontal position; for if syncope then occurs, we have no resource from change of posture.

---

VI.

PART I.—The Principles and Practice of Obstetric Medicine, in a Series of Systematic Dissertations on Midwifery and the Diseases of Women and Children. By Davrd Davis, M.D. &c. Quarto, with a Plate.

As this first part only contains the introduction of the author, and some observations on the anatomy of the female pelvis we can do little more than announce the publication, till we see further specimens of the performance. It is the usual practice of lecturers on midwifery to discuss the whole of the obstetric department at the beginning, or at a very early period of their course, taking up the diseases of women and children successively afterwards. The author flatters himself that he has adopted a more consecutive and philosophical arrangement of his matter.

"The peculiar diseases of women are principally affections of organs which are proper to the sex. Of such organs there are diseases of structure and derangements of function. To obtain an accurate knowledge of either of these varieties of morbid condition, it is manifest that an acquaintance must be previously possessed with the structure and functions of the same organs in their healthy state. In accordance with these views, the author has chosen for himself an arrangement which he believes will enable him to treat of all the subjects, which he will have to discuss, in their most obvious connexion and natural juxta-position under their respective heads of, Structure, Functions, and Morbid States both of Structure and Functions."

The second chapter is on the pelvis, and we take the following remarks on pelvimeters as a specimen of the work, so far as it has yet gone. After giving a pretty full account of the various pelvimeters invented on the Continent, Dr. Davis observes :—

"In this country artificial pelvimeters have been very seldom used, and, perhaps, never exclusively depended upon ; whilst, indeed, there is not one diameter, nor any portion of the cavity of the pelvis, which cannot be much more accurately ascertained by the natural pelvimeter, the practitioner's hand, than by any artificial means whatever. The length of the conjugate diameter may, at all events, be most accurately determined in this way. see pl. iv. fig. i, ii. The patient being placed on her left side, the medical attendant's fore-finger is to be carried up to the promontory of the sacrum. Whilst the point is made to rest on the sacral projection, some inferior portion of it will cross the arch of the pubis, the apex of which it may be made more or less distinctly to feel. This part of the finger should be accurately marked with the nail of the index finger of the practitioner's other hand. The intermediate distance between the part of the finger so indented and its point resting on the promontory of the sacrum, will of course be the measure of a line drawn from the promontory of the sacrum to the apex of the arch, the thickness of the symphysis pubis inclusive. Making an allowance for this thickness, and for the greater length of the line thus obtained, by reason of its greater inclination, than of the conjugate diameter, the length of the latter line, the conjugate diameter of the brim of the pelvis is determined with considerable accuracy. Should the practitioner's index finger prove too short to reach the promontory of the sacrum, presuming on its being of the ordinary length, and on its being passed up in a proper direction, it would then be a matter of generally safe inference that the pelvis was of sufficient capacity in the direction of the conjugate diameter.

Inasmuch, however, as a greater degree of precision might be desirable in some doubtful cases, it would be quite easy for the medical attendant to obtain it by carrying up his index and long finger together, so that the latter might be placed upon the promontory of the sacrum. He would then have to indent the nearer part of his index finger as in the former case. The distance between the part so indented of the index and the point of the long finger resting upon the sacrum would give the length of the conjugate diameter, together with the allowance of half an inch for the greater length of the line actually measured, and the thickness of the symphysis pubis. The distance so obtained would be easily determined by applying a common rule to it.

But the conjugate diameter may by possibility be of sufficient length, and yet the space on either side of it might be too contracted to admit the head of a well-grown child to enter into the pelvic cavity. The fact of this malformation may also be ascertained with tolerable accuracy by the hand. For this purpose the whole of the hand is to be introduced into the vagina and carried up edgewise, *i. e.* with the index finger in front, and the little and ring fingers behind, to the lateral portions of the pelvic cavity; so that the points of the fingers shall rather more than clear the brim. Should there be found sufficient room on either side of the diametrical line for the hand so introduced to maintain the parallelism of its fingers, *i. e.* to lie there without being forced to ride over each other, then it would be matter of conclusion that with sufficient space in the middle, added to the ordinary space on the other side, there would be found ample room for the descent of the child's head into the general cavity. The practitioner then would have to pass his hand along the other side of the pelvis, and subject its dimensions to the same test. If, indeed, the hand was carried up along and nearly in contact with the lateral parietes of the pelvis, and room was found there even for THREE FINGERS to lie together in moderate parallelism, the conjugate diameter being at the same time of sufficient length, it would be competent for the medical attendant as a general rule, to infer favourably of the dimensions of such a pelvis."

An excellent lithographic plate, containing various views of the pelvis, is prefixed to the fasciculus. The work promises well, from the talent of the author, and the cheap mode of publication.

---

VII.

### ON ONE OF THOSE AFFECTIONS DESIGNATED MIGRANE, (MEGRIM,) OR HEMICRANIA. By M. PIORRY, M.D.

THE foregoing subject occupies some twenty pages of M. Piorry's late work on pathology, diagnosis, &c. from which we have already made some extracts in other parts of the Journal. The author observes that, under the term hemicrania, authors have arranged several different disorders. Chaussier and his followers have pronounced it a neuralgia of intermittent or continued character, and of greater or less intensity. The author agrees with this opinion; but observes, that hemicrania cannot always be considered as a facial neuralgia. It differs, he remarks, materially from tic douloureux, and also from the pains occasioned by carious teeth. If, indeed, says he, we understand by the word hemicrania, a pain seated on one side of the head, almost all neuralgia of this part must come under the designation, since few of them attack more than one side of the body, or pass the median line. But if, by hemicrania, we mean a specific affection, having its seat in or near one of the eyes—differing from all the other neuralgiæ—followed by sickness and generally relieved by vomiting—ceasing after a single paroxysm, not to return for a considerable time in general—then we must separate hemicrania from the other neuralgiæ with which it has hitherto been confounded, in order to study its symptoms, its signs, and its treatment. The author affirms that he has paid great attention to this disease —and thinks that investigation of it throws some light on the neuroses in general.

The complaint in question is conceived by the author to be a neurosis, or rather neuralgia of the iris, which, at first bounded to that membrane, or, more properly speaking, to its nerves, extends to a number of other nervous branches, and is characterised by disturbance of vision, succeeded by pain in the eye, or on the surface of the cranium, by sickness, and by vomiting. This ophthalmic neurosis is observable among people whose sight is weak, to whom strong light is disagreeable, and dark rooms pleasant—who study and write much—who lead a sedentary life—and among workmen who are much occupied with the inspection of minute bodies.

On the other hand, we rarely see this complaint in people who lead an active life in the open air—who are habitually exposed to a strong light—and who do not exercise the eyes much. It occurs chiefly, according to the experience of the author, under two opposite conditions of the stomach—a state of too much repletion, and too great abstinence. In people, in these conditions, a very slight exertion of the eyes will often bring on the ophthalmic neuralgia. A physician of the author's acquaintance generally experienced an attack of this complaint every time that he read a lecture on medicine. Whenever he left off lecturing, or at least the reading of his lectures, he ceased to have the hemicrania; and whenever he resumed the avocation, the disorder returned. It was remarked that these lectures were delivered on a full stomach directly after dinner.

The hour of attack, however, is not confined to any particular period—sometimes immediately after the application of the exciting cause—sometimes not till after several hours, but generally within the 24 hours. At the moment of invasion, the sight becomes less clear, and there appears a kind of black speck in the centre of the eye, which gradually enlarges and spreads to the other parts of the organ, still partially surrounded by the arc of a luminous circle, of different colours in different individuals. After a time, this dark centre and sparkling circle begin to grow less distinct, and at last break up and disappear, with return of vision. These phenomena rarely take place, except in one eye. Thus far there is no pain experienced; but only a kind of stupor, with some derangement of vision and heaviness of head. But after a longer or shorter interval, some darting pains are felt in the eye and temple, and the least pressure on the ball of the eye causes much suffering. The patient complains that the globe of the eye feels too full, attended with pulsation of a dolorous kind. These sensations are not uniformly pungent, but remit and exasperate, like colick or spasmodic pains in other parts. The duration of these attacks varies from some hours to two or three days.

Meantime the senses of hearing, tasting, and smelling are more or less deranged with that of sight. The eyelids become red and tumefied—the access of light is insufferable—the least noise offends the ear—and the taste for food is quite perverted. The sensorial functions are undisturbed; except that there is a greater tendency to sleep than usual.

Such are the phenomena of cases the most simple; but very frequently the stomach participates in the complaints of the eye. Soon after the ophthalmic symptoms commence, eructations from the stomach take place, followed by some nausea, and even by vomiting of the food lately taken, or, if empty, of glairy mucus. In severe cases the stomach is not the only organ which sympathizes with the eye. Often one side of the tongue or of the face; or one of the upper or lower extremities experience a kind of painful tremor or vibration, like that which is felt after striking the cubital nerve, at the elbow, against some hard body. In general, the heart, lungs, and intestinal canal remain free from any morbid affection. A restorative sleep usually terminates the paroxysm, after several hours, or two or three days' duration. A heaviness of the head is felt for a day or two after the cessation of the hemicrania. The recurrence of the malady is uncertain; and generally only when the exciting causes are strongly applied. Our author knows a female who has six months' interval between the attacks, provided she does not read within two hours after taking food. If she uses not this precaution, she is sure of an attack immediately after her transgression. In certain individuals the attack is periodical, returning every eight days, every month, or every two or three months, with considerable regularity. In others, there is no fixed period for relapse.

The prognosis in this curious complaint, is generally favourable, as far as life is concerned; but if the complaint proves rebellious, it renders life miserable. When it fails to be cured, the paroxysms return at shorter and shorter intervals, till life becomes one scene of suffering. Our author has not been able to find any information respecting the pathological anatomy of hemicrania; but suspects that the scalpel will not reveal any lesion of structure in the brain or membranes to account for the phenomena. This neuralgia, he imagines, is too fugitive, subject to too many remissions or intermissions, to leave organic traces that might be detected by the eye. He justly observes that the dissecting-room is not the only place where we may study pathology. The sick-room will often afford us much useful information in this respect. An examination of the eye, during the attack of hemicrania, shews the pupil strongly contracted, and consequently the iris put upon the stretch, with redness of both palpebræ. From these phenomena M. Piorry concludes that, in hemicrania, an exciting cause acts on the retina and iris—the nervous action is modified—a kind of struggle takes place, evinced by oscillations and vibrations, with the luminous circle, dark spots, &c. before alluded to. In time, the fifth pair of nerves participate in the morbid action, and ultimately other organs and parts with which the fifth pair communicate.

### TREATMENT.

Our author considers himself as very successful in the treatment of this painful malady. His first indications are to arrest the development of the series of symptoms constituting hemicrania—and to mitigate its accessions, (calmer ses accés.) It is at the moment of its commencement that the course of the malady is most easily checked. At this period all causes of excitation in the optic nerves and tissues should be removed. The patient is to be completely excluded from light and noise. This abduction of all stimulus will sometimes induce early sleep, and check the paroxysm. It is at this early period that the application of belladonna has occasionally succeeded in preventing the pain. The author and his colleague, M. Trousseau, cause the remedy to be rubbed on the temples, with the greatest success. M. Piorry dilutes the belladonna with a little water so as to form a kind of syrup, which is rubbed on the palpebræ as well as on the temples. The author uses only a very small quantity of the remedy, from one to three or four grains. He avers that he hardly ever fails to check the paroxysm by this means. It is to be borne in mind that, on the succeeding day, after the application of the belladonna, the pupils will be greatly dilated, and vision disturbed. But this effect and inconvenience are only temporary. The author has not employed belladonna internally. He has used opium, but with very indifferent effects. M. Piorry observes that the march of hemicrania may often be arrested by raising excitement in the stomach, by means of stimulants, as wine, spices, food, &c. A very smart stimulation to the feet will sometimes have the same effect. In case of failure, each symptom can only be combated by the most probable means. Quietude and darkness—cold applied to the eye affected—vomiting by means of large ingurgitation of warm water, will mitigate the pain, and somewhat curtail the paroxysm.

The prevention of a return then becomes the great indication. The causes already enumerated, are to be avoided, and especially all exercise of the eye in reading during the operation of digestion. After this process is finished in the stomach, the patient need not fear to engage in study or other exercise. Exposure to a strong light, however, and sudden transition from a dark to an illuminated room are dangerous. Sometimes a local plethora predisposes to hemicrania, then abstraction of blood is proper. On the other hand, where the patient leads a sedentary life, and is debilitated, we ought to prescribe nourishing food, which is the best of all tonics. Great attention to the bowels is necessary, since constipation often renews the attack. It is remarkable that the author appears to have had but little experience of the efficacy of quinine in this complaint—and of arsenic he makes no mention at all. Yet these are the most potent of all remedies in the disease under consideration. We have met with the complaint very often—indeed, it is by no means unfrequent in this metropolis, among artists and others who lead a sedentary life, using the eye much, and the muscles of the body little. In these people, we have first cleared the bowels, and then given a sudorific at bed-time, with a good dose of colchicum and Batley's liquor opii sedativus. After this the quinine, arsenic, or both united, have seldom failed to put a sudden stop to the complaint. Quinine in small doses, for some weeks afterwards, is necessary to prevent relapses.

---

### VIII.

Lectures on Anatomy: interspersed with Practical Remarks. Vol. III. By B. B. Cooper, F. R. S. Surgeon to Guy's Hospital, Lecturer on Anatomy, &c. &c. &c. Royal 8vo. pp. 300, 5 plates. London, 1831. Price 15s. bds.

Two volumes of this very useful work have been already published, and noticed in this journal. The third is now before us, and it does not derogate from the reputation acquired by its predecessors. So far as we can see, this system of anatomy is likely to prove the most complete, comprehensive, and generally useful of any at present before the public. Mr. Cooper appears to be taking great pains to make his Lectures as useful to students as possible, and we do not doubt that those endeavours will be met by corresponding success. We cannot pretend to offer a formal review of a treatise on anatomy, but we may state that the practical remarks contain valuable information to students, whilst the general anatomy is copious, the physiological portion enriched with all the current knowledge of the day. As an instance of the manner which physiological subjects are treated, we are tempted to give rather a long extract; it is on the physiology of the human voice.

"The voice is produced in the larynx by its mechanical operation upon the air passing through it, much in the same manner as sounds are emitted from wind instruments; these sounds are variously modified, not only by the larynx itself, but in the direction they are made to take in their progress through the mouth and nasal passages, by the appropriate motions of the lips, lower jaw, tongue, pharynx, and fauces, as well as by the length of the trachea.

Speech is an acquired faculty, resulting from the appropriation of certain sounds emitted by the voice to express our ideas. The sounds constituting speech are infinitely numerous, as may be seen in the various languages and dialects of different nations.

The sounds of the voice, and their innumerable modifications, may be considered under three heads; natural, imitative, and musical sounds.

Natural sounds belong to all animals that have lungs; and may we not add the 'voice of the cricket,' though made by the motions of its wing and leg, the hum of bees, and various other sounds produced by insects.

The cry is the earliest natural sound, and establishes a remarkable instinctive intelligence between parent and offspring. Thus it readily expresses pain and fear, and will incite the almost defenceless hen to face the ferocious mastiff, in aid of her little ones in danger. The cry expresses the most simple wants and natural passions; and this sort of language is found in most animals. In man it belongs to all ages and states; the infant and the aged, the savage and the civilized, the idiot and the person born deaf, all have the faculty of crying. It expresses many of our most vivid feelings; thus there is a cry of rage, of fear, of pleasure, and of pain.

Imitative sounds form the basis of all languages; hence speech is never acquired without the aid of the sense of hearing. Persons born deaf are also dumb, so far as the faculty of speech is concerned; for the simple reason—because the ear is unable to appreciate the sounds which are employed in language to express objects and ideas. For the same reason, a person who hears badly, is often inclined to speak high; or, having a false ear, has also a false voice: and those born deaf, who have been taught artificially to speak, have voices hoarse, dull, and with unequal inflections.

Young children begin by imitating the sounds they hear, in a very imperfect manner, being only capable of expressing those which are the most simple. In their early attempts they frequently substitute vowels, and sounds easy of expression, for names which are in any way complicated or harsh; so also in their first attempts to construct a sentence, their errors are all simplicity, and are regulated only by careful education, and the force of correct examples.

Nothing proves speech to be the result of imitation, regulated by civilized education, more than the existing varieties of language among different nations, as well as the peculiarity of dialect in different parts of the same country.

Rhetoricians have entertained various opinions respecting the origin of languages, which would be superfluous to consider here, as indeed they have been in general too speculative to have led to any useful conclusion.

The modifications of the voice are expressed in letters; and these, variously combined, form words, which are the signs of ideas, and the component parts of language.

The letters or alphabet are pronounced by varying the mode in which the voice is emitted: some letters, being quite simple, require hardly any action beyond the larynx itself: others require the motion of the tongue acting with its tip, middle, or base; others of one or both lips; while for the production of some, the air is made to pass the nasal cavities. Indeed, a clear pronunciation of any sound is not made when either from disease, or artificially, the nasal cavities are closed. The air, in its passage through the mouth only, appears to be arrested, and to impart a thickness to the voice; or, what is improperly termed, speaking through the nose.

The letters have been divided by grammarians into vowels and consonants; but, in a physiological point of view, we rather consider the mechanism by which they are pronounced. Thus the letters in all European languages, consist of those which are purely vocal, and are pronounced with very little effort; these are *a*, *e*, *i*, *o*, *u*, named vowels, and proceed entirely from the larynx, subjected however to numerous modifications, depending on the force with which they are uttered, producing long or short accents. So also we have, as enumerated by Majendie, *a* very open, as in *hall*, English; *â*, in *hâle*, French; *a*, *é*, *è*, and *e* mute, French; *i*, *o*, open, Italian; *o*, *eu*, *u*, French; *u*, Italian. Other vocal letters are *b* and *p*, labial; *d* and *t*, dental; *l*, palatine; *g* and *k*, guttural; and *m* and *n*, nasal consonants. The other letters are *f* and *v*, *th*, English; *s* and *z*, *ch*, *j*, *r*, *h* and *x*, Spanish; χ, Greek; and are produced chiefly by the air being forced against the sides of the mouth, and directed by the tongue, either by its tip, middle or base: *v* and *f*, by the lower lip and upper teeth: *r*, in English, has a much softer sound than in French, where it is pronounced by a vibratory motion of the tongue against the roof of the mouth.

Words are compound sounds, formed by the combination of letters; and being arranged according to the rules of grammar, constitute a language.

Languages are more or less harmonious, in proportion to the number of vowels or simple tones which it contains; thus the Greek and Latin, among ancient languages; and among modern, the Russian, Italian and Spanish are more agreeable than those of teutonic origin, as the French, English, German, Dutch, Sweedish, Danish, &c.

Speech, considered as the means of communicating our thoughts, is the organ of our intellectual faculties: and herein constitutes the great superiority of man over the rest of the animated creation. He alone can express his feelings in appropriate language; can accumulate knowledge in the storehouse of his memory; can search for causes, and distinguishing effects; and, above all, can judge and reflect upon the past, the present, and the future.

The third class of sounds produced by the voice is musical sounds, or vocal harmony.

Singing requires greater exertion than is used in speech, and the organs of voice are called into more varied as well as more powerful action. Thus the trachea is lengthened or shortened, by the stretching out, or drawing in of the neck; inspiration is accelerated, prolonged, or slackened; the larynx rises or descends; while the glottis is made to enlarge or contract, either by sudden and quick, or slow and gentle motions. Singing, therefore, consists of modulations of the voice; imitating, with varying rapidity, the different degrees of the harmonic scale; passing from grave to the acute, and from acute to the grave, in sounds which are appreciable by the ear, and regulated by the rules of harmony.

The powers of the human voice are very extensive, and vary remarkably at different ages and in different individuals. The characters of voices are distinguished by the names grave, acute, high, counter, tenor, base, falsetto, &c. We have already compared the voice to a musical instrument, in which the air is made to vibrate as in wind instruments: such, for instance, as the clarionet. In this a thin plate is made to vibrate, intercepting and allowing by turns the passage of a current of air, by which sounds are produced: this structure, termed the reed or anche, is fixed to a tube or body. If the plate is long, the sounds are grave; if short, acute, owing to the greater rapidity and strength of the vibrations in the short than in the long plate. The tube or body has no other influence on the sound produced, than upon its loudness or intensity: those which produce the loudest sounds are of a conical form, increasing in width towards their outer end: if the cone is inverted, a dull sound is produced; but if two equal cones are placed base to base, and adapted to a conical tube, as in the flageolet, the sound acquires fulness and power.

The larynx has been compared to different musical instruments by different physiologists. By Galen, to the flute; Dodart, to the horn; Ferrein, to the reed instrument; but it is considered at the present day to partake of the powers of both wind and stringed instruments, and capable of being regulated by vital action. The quantity as well as the force with which the air is propelled through the larynx, variously contributes to the loudness of the voice: these circumstances are regulated by the size and muscular power of the chest, the volume or size of the larynx, and the power of its proper muscles. In singing, the larynx acts the part of the reed, while the trachea answers to the tube: thus, in running up or down the whole scale of sounds, the neck and trachea are visibly shortened or stretched out.

Acute and grave sounds are produced by the motions of the rima glottidis, regulated by the tension of the vocal cords and their vibrations, as they are variously stretched by the muscles in the peculiar mechanism of the larynx. In grave sounds, Magendie observed in some experiments on dogs, that the air passed out through the whole length of the glottis, the ligaments vibrating in their whole extent. In acute sounds, only the posterior or arytenoid portion of the ligaments vibrated; and in proportion to the diminution of the opening of the glottis, so was the acuteness of the sound. The arytenoid muscles are those which principally produce these acute sounds, as is proved by the division of the superior laryngeal nerve which supplies them; and when divided, they establish a grave tone. It is therefore readily seen how much depends upon the consent of the muscular actions of the larynx, and the consent of the will, in exciting a due degree of tension and relaxation. A sound is readily produced in a larynx when blown into out of the body, and which will be similar to the voice of the animal to which it belonged, but different from that produced during life, in consequence of the want of that regulation which is produced from the tension of the vocal cords during life. Independent of these sounds, each individual has a particular tone of voice, which is readily detected by those who are accustomed to hear it. The same thing occurs among animals: in the same flock of sheep, each ewe and its own lamb are attracted by each other's bleating, and not by those of others.

The voices of women, children, and eunuchs are more acute than those of men, from the comparative smallness of the larynx: it becomes weaker also during illness, from the loss of power in expiration.

The whisper is produced in a similar manner by a weakened action, in which the sound is not more audible than that produced by the passage of the air in forced respiration."

We entertain a very favourable opinion of this volume.

---

### IX.

MAINGAULT'S ILLUSTRATIONS OF THE DIFFERENT AMPUTATIONS PERFORMED ON THE HUMAN BODY; REPRESENTED BY PLATES DESIGNED FROM NATURE; WITH ALTERATIONS AND PRACTICAL OBSERVATIONS. By WM. SANDS COX, Lecturer on Anatomy and Surgery at the Birmingham School of Medicine, and Surgeon to the General Dispensary. Folio, 8 lithographic Plates. London and Birmingham, 1831.

So many plates, etchings, lithographs, and copper-plates, bestrew our tables, that we scarcely know whither to turn to avoid them. Every day sees the birth of some production of this description. If the public buy them all, then happy are the authors: if not, the spectacle of unsuccessful candidates for fame or pelf, has not the effect of deterring others from entering the lists. Each, when he publishes *his* new work, declares that he does so to remedy an inconvenience generally felt, to supply a desideratum universally acknowledged. Yet still inconveniences and desiderata seem rather to multiply than diminish. The object of the present *porte-feuille* is the delineation of the several amputations of the upper and lower extremity. The execution is tolerably good, the aim is deserving of commendation. We cannot convey by words an idea of the lithography; we need not attempt to notice the letter-press, which is explanatory, and little more. The surgical student will judge whether he can afford to purchase the work, to obtain the professional information which it offers.

### X.

ELEMENTS OF SURGERY. By ROBERT LISTON, Fellow of the Royal Colleges of Surgeons in London and Edinburgh, Surgeon to the Royal Infirmary, &c. London and Edinburgh, 1831.

WITHIN the last few years, compilations, systems, manuals, and guides of all descriptions for the use of students, young and old, have multiplied beyond enumeration. If mind is on the march, it is encumbered, like our English armies, with a monstrous quantity of baggage and camp-followers. Yet, amidst much useless matériel, there is also much that is necessary, serviceable, and valuable. Our young aspirants to professional fame have only to climb the ladder, whose spokes are numerous and strong;—their forefathers were compelled to clamber to the summit on deficient steps and frail support.

Mr. Liston has added his powerful assistance to those who are engaged in the laudable employment of disseminating surgical knowledge. Those who read may now acquire, for a sum which even poverty might spare, such learning as books can afford, such knowledge as may qualify them for entering on the observation of Nature herself. The cry has been that books are useless—the cry is, that they are omnipotent. They are neither. Practical knowledge, without book-learning, is barren, egotistic, fraught with prejudice, prone to unreasonable doubt, and too-yielding credulity. Book-learning, without practical knowledge, is vain, confident, subtilizing in discourse, in practice timid, wavering, versatile, and destructive. All men now confess the utility of books, and many perceive the true extent of their benefits. But we are not writing an essay on the times.

We should have taken notice of Mr. Liston's Elements of Surgery sooner, had we not been accidentally prevented from perusing them. As the work is thus avowedly elementary, it is not adapted for review or extended notice in a journal which is avowedly not so. We may offer a sample or two of the matter and the style, in order that our readers may be enabled to form some conjecture as to what they may expect, if disposed to purchase. The following observations on the affections of the nose will suit us.

" *Of Inflammation, Abscess and Ulceration of the Nose, and Cavities connected with it.*—Inflammation may be excited in the nose by external injury, as a bruise, or fracture, or displacement of the bones. The acute symptoms are swelling and discoloration of the integuments, turgescence of the schneiderian membrane, which covers the septum naris and the turbinated bones, and consequent obstruction to the passage of air. Unless active measures are pursued, abscess follows, with greater swelling and obstruction; and extensive loss of substance, with deformity, may ensue. Unless the acute symptoms, the short duration of them, and the rapid supervention of tumour be considered, the swelling may be mistaken for polypus.

The septum suffers more than other parts of the nose, from the concussion produced by a blow, and is in general more seriously affected by the morbid action which is induced. Matter is effused beneath the membrane, in one or both sides, usually in both, and tumours are thereby formed, which project into the cavities of the nostrils; when attentively examined, fluctuation is felt, and if the affection has existed for a considerable time, the abscesses are found to communicate with each other, the septum having been absorbed or necrosed at one or more points.—An individual received a severe blow over the extremity of the ossa nasi, and a slight wound was produced. The breathing soon became obstructed, by swelling in the nostrils, and great pain in the part was complained of. A large tumour formed on the septum, and completely filled the cavities; it was opened, and a great quantity of matter evacuated. The septum was destroyed by ulceration to a considerable extent, and a slight falling down of the middle of the nose followed. Such cases are of common occurrence.

Independently of any vice in the constitution, *ulceration* of the nostrils may be induced by injury, and proceed until great ravages are effected, if the treatment be not properly conducted.—A young gentleman playing at ball, was struck accidentally on the nose with the flat part of his companion's hand. Inflammation took place, externally and internally, and the passage of air was obstructed; abscess formed, and the matter was evacuated spontaneously; extensive ulceration ensued; the cartilage and bone became affected, portions of them separated, and a bloody fetid sanies flowed from the nostrils. All the cartilaginous, and part of the bony septum were destroyed; the morbid action ceased after having continued for a long time; but the organ was curtailed, sunk on the face, and altogether much deformed.

The alæ, as well as the septum, may suffer from external injury, indeed the whole cartilaginous part of the nose may be destroyed.

Incited action must be subdued by abstraction of blood from the external parts or from the schneiderian membrane, leeches being applied in sufficient numbers, and repeated. Should suppuration not be prevented, the abscess, particularly when internal, must be early opened: the surgeon is to blame, if the patient, having been under his care from the first, sustains any deformity. If abscess has formed on both sides of the septum, each must be opened freely; afterwards hot fomentations are to be used, and the cavity should be frequently cleansed by the injection of a bland and tepid fluid.

*Intractable ulceration* of the nostrils, is often induced by trifling irritations or injuries in constitutions, either originally unsound, or rendered so by imprudent conduct; slight blows on the prominent part of the organ produce swelling with discolouration, and that is followed by abscess and ulceration. Internal ulceration is frequently caused by the continued use of snuff, or the presence of other irritating matters,—by irritation communicated from diseased gums or alveoli, or from decayed or crowded teeth, particularly the incisors of the upper jaw—by stumps in any part of the mouth, or the pivoting of artificial teeth on them—by introducing the dentist's perforator, with a view of destroying the nerve of a tooth. I have seen ulceration, and loss of substance, arising from each and all of these causes.

The ulceration occasionally commences, even in young subjects, in a wart or fissure on the integuments of the nose or upper lip; it thence extends to the alæ and floor of the nostrils; the cartilages, and even the bones, are destroyed; the discharge is thin, acrid, bloody, and fœtid, and the action is with much difficulty controlled. The disease is met with of various degrees of severity and malignancy; it may cease spontaneously, may appear to be arrested by constitutional and local treatment, or, resisting all means employed against it, goes on consuming portions of the face, both hard and soft; destroying the nose, lips, and eyelids, and ultimately the bones in their neighbourhood. Horrid cases are occasionally met with, in which scarcely the vestige of a feature is discernible—the patient is nourished, and life, which cannot be enjoyed, is protracted by food conveyed over the root of the tongue, through funnels or tubes. *Noli me tangere*, and *lupus*, are names applied to the advanced stages of the disease.

*Ozœna*, which denotes the internal ulceration of the nose, or rather the discharge indicating such, is generally of long continuance. The discharge is at one time profuse, at another scanty; sometimes is ceases almost entirely, but the accompanying fœtor, of a most disgusting nature, is still perceptible on approaching the patient, or coming within the influence of the air expired over the diseased surface; the stench is particularly offensive when portions of bone are separating. The bones may die either from inflammatory action in them running high, or from being uncovered and deprived of support by ulceration of the investing membrane. In many cases, the disease is not arrested till the cartilaginous and bony septum, the turbinated bones, the hard and soft palate, and frequently the alveoli, are completely destroyed. The patient, if he live, is in a miserable plight;—his countenance is deformed and ghastly: the situation of the nose is occupied by a large dark and foul sore; the discharge is profuse and weakening; the expired air is as a pestilence to himself and those around; speech is almost unintelligible; breathing is difficult; the strength is gradually exhausted, and the spirits sink under the harrowing impression of misery. All these ills result more frequently from the injudicious employment of mercurial preparations than from any other cause. In almost every instance, the predisposition to such frightful ulcerations has been induced by the use of mercury, and can readily be traced to it. Exposure to atmospheric changes, during or after the exhibition of mercury, may render the mucous surface and the coverings of the bones more susceptible of the disease; that medicine may be given with the utmost precaution, but for long after the constitution cannot shake off its influence; and too frequently more of the poison is administered for disease produced by it. Ulceration of the tonsils, and other parts in the fauces, often co-exist with disease of the nostrils.

Ulceration of the nostrils is arrested with difficulty. It cannot be expected to cease till dead parts have separated, become loose, and fall out, or are removed by art. Portions of the bones, forming the floor of the nostril, can often be removed, when dead, through ulcerated apertures in the palate; whilst others are brought away through the nostrils, there being generally sufficient space allowed for their discharge—the nasal cavities being laid into one by destruction of the columna, and more or less of the septum. Occasionally the ossa nasi, or parts of them, escape through an opening in the superimposed integuments; sometimes they cannot be discharged otherwise, as in the following case:—Matter had come to the surface over the nasal process of the frontal bone, an incision was made for its evacuation, sequestra were found loose, and some extracted; one was pushed down with the view of pulling it through the nostril, but this was found closed from the effects of small-pox.

Various applications to the ulcerated cavities are employed. Injections of spirituous and aromatic lotions are used to wash away the discharge and correct the fœtor, as diluted tincture of myrrh, tincture of aloes, a lotion of the sulphate of zinc, solutions of the chlorates of lime or soda, &c. Applications, soothing or stimulant, are made to the exposed sores according to their appearance and disposition. When the ulcer is of an angry and irritable aspect, it is to be touched lightly with the nitrate of silver, in substance or solution, and then covered with a bread and water poultice. Fowler's solution of arsenic is useful in some cases, when the object is to clean or destroy the surface; this is also effected by a slight application of the potass. Black wash sometimes agrees well, as also a liniment of olive oil and lime-water, with citrine ointment (three parts of the former ingredients to one of the latter), or the sulphate of zinc lotion. When the sore is very indolent, showing no signs of granulation, it may be touched occasionally with spirit of turpentine, either pure or combined with alcohol, and afterwards covered with an ointment composed of ung. ceræ and spir. terebinthinæ; under this application ulcers often heal, after having resisted all others. But nitrate of silver applied gently, and repeated at the interval of two or three days, will, in the majority of cases, be found the most efficient remedy. Constitutional treatment must not be neglected. When the disease cannot be traced to mercurial action, small doses of the muriate of mercury are allowable when excitement is required. The arsenical solution given internally sometimes produces good effects. In foul internal disease of the nostrils with cachexia, no medicine exerts so beneficial an influence on the general health and local disease, as sarsaparilla, exhibited either in decoction, in extract, or in powder. The solid extract is the preferable form, and is given either amongst milk, in substance as a bolus, or dissolved in water or in distilled aromatic water."

Two parts, or volumes, have been already published, the third and concluding one is "in active preparation." They will comprise a system of surgery, culled from personal experience, and from the recorded practice of the best surgeons. In the second part there is a succinct account of the diseases of the eye. Inflammation of the bursa over the olecranon process, is a frequent occurrence, yet we constantly see it mistaken by practitioners. It is right to put them on their guard, which we may do by a short extract from Mr. Liston's work.

"The *Bursa* over the *Olecranon Process* is liable to enlargement, by gradual accumulation of the secretion, in consequence of habitual pressure on the elbow. The contents are either serous or albuminous, usually the latter, and the swelling is indolent. But acute swelling not unfrequently takes place in this situation, from external injury; then the tumour is formed rapidly, there is heat and pain in the part, and the integuments are discoloured around: in such cases the bursa is filled with pure blood, or with a sero-purulent and bloody fluid. Inflammation of the bursa often follows bruises and lacerated wounds, and is apt to extend to the forearm and arm; causing extensive and deep effusion, great tension of the parts, and severe constitutional disturbance.

In the chronic cases of bursal enlargement, pressure is to be avoided; and by the permanent application of a gum and mercurial plaster (emplastri gummosi—emp. hydrargyri—ā part. æq.)—absorption of the fluid may in general be procured—the swelling disappearing as gradually as it arose. If the collection is large and obstinate, repeated blistering may be had recourse to; and if that fail, a seton may be passed through the cavity. But the last-mentioned practice is sometimes followed by more action than is desirable, inflammation of the surrounding cellular tissue supervening, and abscesses forming, perhaps extensive. When the collection is purulent, a free opening is to be made into the bursa, and the case treated in other respects as a common abscess. If indolent swelling of the cellular tissue, and spongy thickening of the synovial surface of the bursa, remain after incision, the caustic potass should be applied. In extensive and acute inflammation spreading to the surrounding parts, free incisions are required, along with proper constitutional treatment."

We are induced to notice Mr. Liston's treatment of dissection-wounds, for a reason which we will state, when we have put our readers in possession of the treatment in question.

"Wounds received during dissection occasionally have unpleasant consequences from the absorption of putrid animal matter. The absorbents leading from the wounded part become swelled and painful, and in slight cases there are shivering and general indisposition for some days. The more violent symptoms arise from examining bodies which are rather recent, and in which putrefaction is just commencing, and very frequently from inspecting the bodies of females who have died of puerperal diseases. The absorption may take place from punctures made by scissors, the point of a knife, or spiculæ of bone, or from old scratches, or chops by the side of the nail or on the hand. There is little or no danger from an open and bleeding wound, as by the flow of blood the part is completely cleaned; it is only from slight punctures that untoward symptoms need be apprehended. Effects similar to those resulting from wounds in dissection often occur in nurses and others who have pricked themselves with pins while washing foul clothes. The symptoms already mentioned are soon followed by others more severe: shivering continues, and the patient is seized with vomiting; the part affected, and often the greater part of the arm, become red and much swollen, and the cellular tissue is infiltrated with serum; abscesses form at various points, often in the axilla, and purulent matter is diffused throughout the unhealthy cellular tissue, which in many instances sloughs, and gives rise to extensive sores. Typhoid symptoms soon appear, and in the more aggravated cases speedily prove fatal. When such local and constitutional symptoms arise, it will generally be found that the patient was of a broken-up constitution previously to the infliction of the wound; did they solely depend on the inoculation of virus, they would be of very common occurrence, considering that wounds are so frequently received during dissection; but it is seldom that any unpleasant symptoms follow such an accident. In all cases, however, it is prudent to adopt measures in order to prevent absorption of the virus. With this view the wound is made to bleed by means of pressure or suction, and by the latter method the exposed surface is most effectually purified; afterwards nitrate of silver may be applied to deaden the surface, and protect it by an eschar. If such means be unavailing, the after symptoms must be encountered as they appear, local inflammation subdued, tension relieved, abscesses opened, sloughs removed, &c. General bleeding is seldom admissible, but purgatives and antimonials will prove beneficial at the commencement; afterwards the strength is to be supported, and, if the patient be much reduced, stimulants are to be liberally administered."

The point on which we would offer a remark, is the recommendation of Mr. Liston to apply the nitrate of silver or some other escharotic to the wound. We have seen this done several times, and in almost every instance with bad effects. Inflammation of the finger or the hand has succeeded, and an angry, irritable, and more or less intractable sore has been left. We believe the best local treatment to consist in the employment of suction, early and careful washing, poultices if any inflammation should occur, and early opening if abscess or merely a pustule should succeed.

It is unnecessary for us to pronounce an opinion on Mr. Liston's work. Its manner will be learnt from the extracts we have given, its matter can only be appreciated by a careful perusal. We trust that it will meet with the success which it deserves.

---

XI.

Observations and Experiments on a new Mode of treating Fractures of the Leg and Fore-arm; especially Compound Fractures. By William Beaumont, M.R.C.S. and Surgeon to the Farringdon Dispensary. Octavo, pp.31. Longmans, 1831.

The author of this pamphlet informs us that, it was after making a series of experiments for the purpose of examining the processes by which union of fractured bone is effected, that he conceived the possibility of greatly accelerating the reparation, and diminishing the danger of compound fractures—except where there was extensive laceration of the soft parts. He conceived that, by maintaining in compound fractures a continued exclusion of the atmosphere from the broken surfaces of the bone, union might proceed, cæteris paribus, as rapidly as in simple fractures, and with almost as little danger. The common experience of surgeons has decided that a compound fracture is infinitely more dangerous than a simple one, and much of the mischief must undoubtedly be owing to the admission or contact of the atmosphere. But we do not consider it as proved, notwithstanding, that this accounts for the whole difference in amount of danger between the species of fracture alluded to. Much must be attributed to the proneness of wounded skin and cellular membrane to inflammation. This is by the way.

The efforts of surgeons have long been directed, in the treatment of compound fractures, to the speedy healing of the external wound. The varieties of apparatus for fractures are endless, the principles of treatment generally recognized at present are simple and few. They enjoin perfect repose, light dressings seldom disturbed, moderate antiphlogistic treatment. The success obtained by these means is far from inconsiderable, yet it must be acknowledged that cases do occasionally terminate unfortunately. Ingenious persons have attempted to improve upon the ordinary treatment, and as the healing of the wound is the great desideratum, they have conceived that by enveloping the limb in an impermeable case, they might obviate the injurious consequences of the breach of continuity in the soft parts. On this principle it is that some surgeons apply varnish to their dressings, that Baron Larrey coats his dressings with a composition and leaves them untouched for several weeks, and that our author recommends the employment of plaster of Paris. By means of a cast composed of this substance the limb must be maintained in one position, the external air is prevented from obtaining access to it, and steadiness and repose are ensured.

"The advantages, then, which I may state this practice to possess over the employment of splints, are, 1st, the maintaining of an unremittingly motionless state between the portions of the broken bones, notwithstanding any moderate movement on the part of the patient: 2dly, the consequent liberty of changing his position, as often as may be necessary to relieve the weariness of a long continuance in the same posture; which change of position, would, in all probability, prevent any ulceration, or sloughing of the integuments of the back or buttocks, when from excessive debility, such an occurrence may threaten to take place: 3dly, the better and complete preservation of the proper length of the limb, in cases where the fracture runs very obliquely across the bones; and also the certainty of preventing any eversion, or inversion, or crookedness. Lastly, there is in thus treating compound fractures, the advantge of so perfectly excluding the atmosphere from the injured parts, as to render the process of union as rapid as in simple fractures. The great danger too, almost invariably attendant upon compound fractures, is also much diminished; if I may so judge from the little apparent suffering of the animals on which I made experiment, as to this mode of treatment. It is seen, moreover, that where there is much constitutional disturbance, the reparation of a compound fracture, or other severe injury, does not proceed with any degree of rapidity, if it proceeds at all; wherefore, as the reparation of the compound fractures, which I have treated according to the method I propose, did proceed with rapidity, one must needs conclude, that the said compound fractures were not accompanied by much constitutional disturbance: so that the mode of treatment, by which the curative processes are most expedited, must also be that by which the danger is most diminished. Now, with the exception of those cases of compound fracture, in which the soft parts are so much bruised, or otherwise injured, as to suppurate, or slough, to great extent; or of those cases, in which a something may happen to destroy life, not necessarily connected with compound fracture, I have not the least doubt, that with the exception of such cases, compound fractures, at least of the leg and fore-arm, may, in general, be made to get well, as rapidly as simple fractures, and, I believe, with almost as little danger."

In order to ascertain the practical value of his method, our author performed some experiments on rabbits; he relates the particulars of two. In the first, the tibia of the rabbit was broken, and both ends of the bone were pushed through the skin, making a large and rough wound. The limb was put up in plaster of Paris, in a way that will presently be described, and the plaster was removed at the end of three weeks and four days. The wound was not quite healed, a small ulcer remaining, through which might be seen a part of the tibia. There was about the ulcer some inodorous, semi-fluid matter, resembling pus deprived of its more liquid parts; a strong and seemingly osseous union had taken place. In two or three days more, the rabbit was able to bear pretty well upon its unsupported broken limb. In the second experiment, the right radius and ulna of a rabbit were fractured, and extending down to the fracture, was made with nitric acid a slough, half an inch long, and one-third of an inch broad, through which slough the broken end of the proximal portion of the bones was pushed. The limb was then put up as in the other experiment. Three weeks and four days after the fracture the limb was examined. An abscess had formed, burrowing from the fracture half way up the humerus, where it had made for itself an opening, through which the bone could be seen separated from the subjacent muscles. Union had not taken place, the wound in the soft parts had not healed, and the limb was again put up as before. At the expiration of eight weeks an unyielding osseous union had taken place, the wound had healed, and the rabbit could bear upon the limb. Such are the experiments and experience by which the plan of treatment is supported. We shall now let our author explain its details.

"Supposing the case of a compound fracture of the tibia, (the fibula being also broken,) supposing the fracture to run very obliquely across the tibia, and the muscles of the limb to have drawn the one portion of the bones considerably over the other: under these circumstances I would advise the following mode of putting up the fracture. The patient should be placed upon his side, with his broken leg and adjoining part of the thigh resting upon a horizontal plane, the outer, the fibular side of the limb being downwards, and the leg bent upon the thigh nearly at a right angle.

A board, about thirty inches long and eighteen broad, which I have had made to place a fractured leg upon during the incrusting of it in plaster of Paris, has a staple fixed at one end, and at the other, a strong piece of iron projecting longitudinally about nine inches, to which is attached an apparatus of pullies, similar to that used to reduce dislocations; two pullies, however, need only be employed, instead of four. In that part of the board, along which it is intended the limb should lie, there are cut several transverse grooves, about three-quarters of an inch deep, and nearly two broad; they are not more than an inch apart, and from three to four inches long. By these grooves, the plaster of Paris, when poured upon the limb, can pass beneath it, and connect below as well as above, the incrustation on one side with that on the other.

A bandage should be put upon the limb from the very extremity of the toes to near the middle of the thigh; which is for the purpose, both of making an equal pressure on the whole of the limb, and also to prevent the plaster of Paris from sticking to, and irritating the skin. The eighteen-tailed bandage should, as usual, be employed, but in order to guard against any derangement of its folds by the force which may be necessary to extend the limb to its proper length, I would advise, as each tail may be laid over its corresponding tail, to sew them firmly together.

In the putting on of this bandage, the greatest attention will be requisite that a moderate and equal pressure be made by every part of it, or, at all events, that the upper part shall not make more pressure than the lower.

Beneath, and also above the limb, that is, along its fibular and tibial sides, should be placed between it aud the bandage, several strips of soft linen or flannel, from three to four inches broad; they will form pads better adapted to the purpose than almost any other.

The bandage should, at first, be put on from the toes to an inch or two above the ankle; and a similar bandage from the head of the tibia to a few inches above the knee. A sort of leather cap which I have had made to fit the ankle, and another to fit the knee, should then be buckled round these parts. The knee is to be made fast to the staple of the board by loops attached to it's leather cap; and that covering the ankle, is to be fixed to the cord passing through the pullies. By these means, extension may be gradually made, and as nearly as possible in the direction of the axis of the tibia: the one portion of which having been drawn as far off the other as should be, the cord is to be made fast in order to retain it so. Having thus extended the limb to its proper length, the distal portion should be placed and held, in a position neither everted, nor inverted, nor bent upon the proximal portion.

The edges of the divided integuments are now to be drawn accurately into contact, and maintained so with strips of adhesive plaster; which with the surrounding skin should then be thickly overspread with some tenacious, unirritating substance, such as will serve to exclude the atmosphere from that which it may envelop: the whole should then be covered with a piece of kid, or chamois leather, the under surface of it having also been spread with the same substance, in order that it may closely attach itself to the parts beneath.*

The eighteen-tailed bandage should now be continued up the leg as far as the knee; which being completed, there should be fixed to the board on which the fractured limb is lying, that which sculptors call a wall, a sort of raised border, from three to four inches in height, placed as nearly parallel as possible to the outline of the leg, about an inch from it, and extending from the toes to four or five inches above the knee.

Sufficient plaster of Paris to fill the hollow between this border and the limb, is now to be mixed with water, and poured into the hollow, so that an incrustation, about an inch in thickness, may cover the whole surface of the limb.†

Previously to pouring the plaster of Paris around the limb, the bandage covering it, and every part with which the plaster can run in contact, should be thoroughly wetted, so that they may not adhere: and in order that the caps buckled round the knee and ankle, may afterwards be withdrawn, their upper

---

* "The following composition I have found to adhere to the skin with great tenacity, and without irritating it: it may also be melted by a very low degree of heat:—

℞. Picis Nigræ, ℥ij.

Ceræ Flavæ, ʒj.

Resinæ Flavæ, ℥ij.

Terebinthinæ, ℥ss. Ft. Emplast."

† "Great care must be taken that the plaster of Paris used, does not contain a large proportion of lime, as such, of course, gives out a considerable degree of heat when wetted. In mixing the plaster of Paris, it should be poured gradually upon the water, and as much of it as the water can liquefy."

parts should be covered with some plastic substance of sufficient depth, that the incrustation may not rise above it.

The reason that I would not have the plaster of Paris attach itself to the bandage around the limb, is that that circumstance would give rise to great difficulty in the removing of the incrustation, should its removal happen to be necessary.

As soon as the incrustation shall have acquired sufficient firmness to allow the limb to be loosened from the board on which it lies, the leather caps covering the knee and ankle should be taken off these parts, and the hollows left by their removal should be filled with plaster of Paris, as well as those deficiencies in the incrustation which will be found on the fibular side of the limb.

Above the knee, where the incrustation terminates, its inner edge should be pared away, in order that it may not press upon, and produce ulceration of the integuments. The space thus left between the incrustation and the skin should be filled with some of the aforesaid composition of pitch, wax, &c. which, by adhering to the skin and also to the incrustation, will prevent the possibility of any communication between the atmosphere and the injured parts.

Should it become necessary to remove a portion, or the whole of the incrustation, it may be done, without disturbing the limb, by making, with a fine saw, three longitudinal cuts from one end to the other of the incrustation, and nearly through it; these cuts should be equi-distant from each other, that is, there should be contained between any two of them one-third of the circumference of the limb. After this, by forcing a broad chisel into the cuts, the incrustation may readily be broken into three longitudinal portions of equal width: the whole can be removed, or the limb may be still supported by that portion which covers its fibular side.

As a long continued state of inaction will cause the muscular part of a limb to shrink, it may be right, after ten days or a fortninght from the putting up of a fracture, to remove so much of the incrustation from the knee to the ankle, (leaving, however, sufficient to retain the limb in *statu quo*,) that one may be enabled to tighten the bandage in those places where, from a diminution in the bulk of the limb, it may have become loose; which being done, the incrustation should again be made perfect."

It is an ungracious thing opposing the proposition of an ingenious man, yet we do not think that Mr. Beaumont's will ever be generally acted on. There are many objections to placing a fractured limb in an unyielding, impenetrable case, and allowing it to remain in it without examination for a long period of time. In the great majority of cases of fracture much swelling follows the injury, and that seldom subsides under the lapse of a week or ten days. We are sure, from experience, that any unyielding bandage or case applied in the first instance must be precarious, if not decidedly injurious; and, if not applied at first where is its advantage? Compound, aye, and occasionally simple, fractures are liable to be followed by low, insidious, and formidable inflammation of the cellular membrane, for which reason it is pleasant to have the power of seeing a limb without disturbing it. This cannot be obtained when an unyielding case is employed. It may be said that this would prevent the occurrence of inflammation, but it is problematical; in the second rabbit experimented on, an extensive abscess diffused itself over the limb. Independent of these considerations, and of others connected with the dressing of the wound, we may venture to express our hesitation in admitting that the position and apposition of the fractured bones must necessarily be so good as Mr. Beaumont supposes.

In reply to these objections we have, practically speaking, the results of the two experiments on rabbits. With respect to such we would observe, that they are far from satisfactory, for it is notorious that the lower animals do not suffer from mechanical injuries to the same extent as man. Again, we are not clear that the rabbits would not have done as well, or better, under other treatment than that actually employed. Perhaps it would be well if surgeons would put Mr. Beaumont's proposal to the test of practice, remembering that a few cases can never determine the value of any mode of treatment.

---

### XII.

### Needle swallowed, subsequently forming the Nucleus of a Calculus, and extracted by operation.*

Needles have frequently been said to take strange and circuitous routes in the human body. Many of the cases must be looked on as apocryphal, but the following is indisputably authentic and trustworthy. It is related by Mr. Logan, of Lanark, and being short is scarcely susceptible of abbreviation.

"On the 29th January, 1830, Anne Wilson, aged 22, a servant girl, of a robust habit and good constitution, residing in the village of Kilcadzow, three miles north of Lanark, having gone to bed, with a large worsted-needle in her mouth, swallowed it during her sleep; next day she had pain in the hypogastric region, when recollecting to have had the needle in her mouth when she went to bed, and as she could no where find it, she attributed the pain to its presence in her stomach. Three days afterwards it had passed the pyloric orifice, and gained the small intestines, in which situation it seemed to have remained for a length of time, as in May succeeding, when she first consulted me, she complained of permanent pain, a little below the umbilicus on the left side; there was at this time no vesicular irritation, and she menstruated regularly, until within two periods of the extraction of the needle. I did not again see her till January, 1831, but in the previous autumn she had complained of great pain in the situation of the bladder, accompanied with frequent calls to micturition, and passed a stone the size of a horse-bean. In January, 1831, the family attendant, Mr. Bouglas of Carluke, sent her to me, desiring an opinion. By careful examination, I found, that the point of the needle had entered the fundus of the bladder, on the left side, and being pushed on, had perforated the parietes of the opposite side, over the right acetabulum. The needle had formed a nucleus for the attachment of a calculus, of considerable size, of a lobulated form, and having its larger extremity towards the orifice of the urethra. At this time, she could not retain her urine above two or three minutes, which was passed with excessive pain, and was deeply tinged with blood, coagulating when cold. Introducing a pair of strong dressing forceps, I grasped the needle by a part free from any calculous incrustation, close to the mucous coat on the right side, and endeavoured, by pushing the whole backwards, to free the point, and bring it into the urethra; but this could not be effected by all the force which it was prudent to exert, on account of the long irritation having thickened the coats of the bladder, and produced such a diminution of its capacity, as to grasp the calculus on all sides. Had this succeeded, the calculus might have been crushed, and the whole expelled without incision.

Being foiled in this attempt, we determined on cutting into the bladder, through the urethra. Eight leeches were applied over the pubis, and a gentle saline purgative administered. On the following day, the patient being secured in the usual manner, with the assistance of Mr. Bouglas, I passed a director into the bladder, previously injected, along which a slightly curved bistoury was run, making a lateral incision upwards, towards the left ascending ramus of the pubis. The finger of my right hand was then introduced, and, pushing gently but firmly upon the calculus, the point of the needle was disengaged by the left hand, and brought into the opening; the forceps were then applied, but owing to the size of the calculus, it could not be extracted without enlarging the wound, which must have endangered the establish-

---

* Glasg. Journ. No. xvi. Nov. 1831.

ment of permanent incontinence of urine. The calculus being of a friable nature, was crushed by the forceps, the needle withdrawn, and the fragments brought away by the scoop. The bladder was then washed out, an elastic gum catheter introduced, and the patient put to bed. In the evening, twelve ounces of blood were taken from the arm, and leeches applied on the accession of any inflammatory symptoms. The bowels were kept open and occasionally glysters administered, accompanied by warm fomentations. The wound suppurated on the 8th day. On the 22d day she was able to sit by the fire and move about a little; she can now retain her urine two hours, and passes it by a voluntary inclination, offering a good prospect that there will be no permanent incontinence."

---

### XIII.

### M. DELPECH ON VARICOCELE.*

THE subject of varicocele has been studied with some attention in this country, especially by Sir Astley Cooper, who has communicated much useful information on its causes, nature, and treatment, in his splendid work on the Diseases of the Testis. Yet still our remedial measures are imperfect, and though we may palliate, we can seldom cure. Varicocele is frequently productive of much inconvenience to the patient. M. Delpech, for instance, asserts that he has known military officers obliged to abandon their profession in consequence. Varicocele also tends to occasion wasting of the testis, partly, we suppose, from the pressure exercised upon the organ and its nerves, but principally from the imperfect circulation which it must create in the spermatic vascular system. We need scarcely allude to the diagnostic marks of the disease, but we may be permitted to mention its more frequent occurrence on the left side, its subsidence under steady pressure whilst the patient is in the recumbent posture, and the return of the tumour when the patient rises, although sufficient pressure is made on the abdominal ring to prevent the egress of a hernia.

With respect to the atrophy of the testicle M. Delpech remarks that it frequently exists to some extent, when the varicocele is not severe, and when careful examination is necessary to detect it. On reducing the varicocele in such cases by steady pressure, the testicle itself seems to disappear also, leaving little but its membranes and vessels behind, a circumstance that, on superficial examination, would hardly be discovered. What is remarkable is this, that when the disease had been relieved by the obliteration of the varicose veins, the size of the testis became gradually restored, and with the size the functions. The pain felt in cases of varicocele is not in the testicle itself, but in the nerves of the spermatic cord. M. Delpech relates five cases in order to display his method of treatment, and to elucidate the history of the malady. We shall briefly mention some of them.

CASE 1. *Varicocele—Ligature of the Varicose Veins.*

J. M., æt. 43, a shepherd, was admitted into the Hospital of Montpellier, with varicocele on the left side, as large as two fists when the patient had been for some time in the upright posture. The pains in the loins were so severe as to prevent him from following his occupation. The testis was wasted, the venereal appetite impaired, and the patient would willingly have submitted to castration. M. Delpech adopted a less severe operation.

The patient being placed upon his back, an incision two inches in length was made near the inguinal ring, parallel to the cord, and over it; the cremaster and its aponeurosis were divided, and the parts composing the cord exposed. The two dilated veins were very apparent, they were raised separately, under each was passed a piece of thick amadou, and over this each vein was secured by a ligature. The size

* Memorial des Hôpitaux du Midi. No. 24.

of the scrotum instantly increased, and the dilated veins formed larger knots below the ligatures. The wound was simply dressed, a poultice and suspensory bandage applied, blood taken from the arm, and the antiphlogistic regimen enjoined. Fever followed, with lumbar pains and enlargement of the scrotum. The fever was subdued by the antiphlogistic treatment, but an abscess formed in the scrotum, and was opened. On the 3d day, the amadou and ligature were removed from the veins. In some of these the blood had become coagulated below the ligature, others were "inflamed and mortified," and had occasioned the abscess. No further bad effects ensued, and on the 20th day the wounds were healed, and the scrotum reduced to its natural size. So early as the 12th day the patient had begun to experience erections, and, on the 15th, the testicle had acquired perceptible increase of size. The patient left the hospital on the 40th day; the testicle being even larger than its fellow, and the venereal desire regained. When seen four years afterwards he was married, had children, and presented no trace of varicocele.

M. Delpech concluded, from this case, that his method of tying the spermatic veins was improper. With our present experience, we need scarcely adduce arguments in support of M. Delpech's supposition.

Case 2.—*Varicocele—Operation—Purulent Deposites in the Liver.*

A young officer of infantry was affected with varicocele of the left side, and suffered so much pain and inconvenience, that he expected to be under the necessity of abandoning his profession. He had formerly suffered from intermittent fever, his complexion was sallow, his form spare. Under these circumstances he applied to M. Delpech, who performed the operation. The incision was smaller than in the former case, and the piece of amadou larger and stronger, in order that the constriction of the ligature might be slighter. On the second day the ligature was cut, and removed with the amadou. The veins formed a full and solid cord, and coagulation had taken place in them below the ligature; the lumbar pains had ceased. On the 12th day the wasted testis had regained some size, and there were erections of the penis; on the 18th, the wound was cicatrized, the cord diminished in volume, and the testicle increased.

On the 24th day the patient was attacked with a rigor and attack of fever; his skin had assumed a slightly yellow tint. On the 25th and 26th days there were two attacks of fever, with rigors, thirst, and depression, but without any local pain or symptom. Quina was prescribed on the 26th, but during that day and night there were three atttacks of fever, and in the evening of the 28th the unfortunate patient died.

*Dissection.* The cicatrix of the wound in the groin was firm. The cavity of the spermatic veins was obliterated, and they were solid and filled with a firm, adherent clot. There was no phlebitis, nor inflammation of the peritoneum. The testicle was in a great degree restored, redder than natural, slightly "infiltrated," but otherwise healthy in structure. The liver was enlarged, with prominences here and there. Under each of these prominences was one or several *foyers* of scrofulous tubercles, of different degrees of consistence, for the most part in a state of softening. A great many abscesses existed deeply in the substance of the liver, and in their neighbourhood the organ was inflamed.

M. Delpech thinks it clear that the patient died of chronic hepatitis. We cannot but suspect, from the sudden access of the symptoms, and from their character, that these abscesses were in reality specimens of those purulent deposites, so frequent after operations and injuries. We acknowledge that, out of the many cases of this description which we have witnessed, we never had an opportunity of observing one in which the original wound was *perfectly* healed prior to the appearance of the visceral abscesses. This may be an argument against their identity with the abscesses in the present case, but we may remark that we are yet too imperfectly acquainted with the laws that regulate their appearance, to consider it a conclusive one, when set in opposition to the symptoms and cadaveric alterations.

CASE 3.—*Varicocele — Operation — Secondary Hæmorrhage—Cure.*

The subject of this case was a commercial traveller, æt, 26, with very large varicocele on the left side. The incision was carried lower than in any of the former cases, and the patient would not submit to the quietude enjoined him. Inflammation and swelling occurred, the amadou and ligature were buried and concealed by it, and on the 6th day it was necessary to put the patient to much pain, and to use many endeavours before these foreign bodies could be removed. Some arterial hæmorrhage followed, and was not subdued by pressure applied upon the part. M. Delpech then adopted the following expedient. A brass pin, of some dimensions, was passed under the artery, and some pieces of amadou being then placed upon the vessel, a waxed thread was twisted under the two extremities of the pin and over the amadou, in the form of the figure ∞. The bleeding was thus stopped, the pin was taken away in three days, and nothing further of consequence occurred. On the fortieth day the patient departed perfectly cured.

M. Delpech was now led to imagine that he might modify the operation with advantage, and in the succeeding case he did modify it accordingly.

CASE 4. A cook, æt. 26, had laboured under varicocele on the right side for eight years. The pain was very considerable, and M. Delpech operated upon him. An incision, an inch in length, was made about an inch below the inguinal canal, in the direction of the cord—the varicose veins were exposed, seized with the forceps, and separated by the nails from the surrounding parts. A strip of amadou, half an inch in breadth, was then passed under the vessels, and its extremities brought out at the wound, and secured there by adhesive plaster. Thus there was no ligature upon the veins; which were merely isolated for a small space from the surrounding parts, and left in contact with the amadou. On the 4th day the veins were red externally, manifestly choked by the adhesive inflammation internally; there had been little swelling or inflammation of the parts around. The amadou was withdrawn. The success of this operation was complete, the cavities of the veins becoming obliterated, the disease cured, and the testis regaining its natural dimensions.

The fifth case need not be particularly related here. A small piece of amadou was employed, it became buried in the wound, peritonitis ensued, and, although the patient recovered, yet troublesome suppuration and a degree of risk were incurred.

M. Delpech thinks the employment of the amadou a great improvement in the method of procuring obliteration of the veins. He thinks it cannot be attended with risk of phlebitis, although section and ligature of the vein certainly are so. We saw fatal inflammation of the saphena vein brought on in an adult, by rough examination only, though it is proper to mention that the patient had formerly had an attack of inflammation of the same vessel. Phlebitis also occurs spontaneously; therefore, we see no reason why M. Delpech's operation must necessarily be unattended with danger. In this country surgeons shew an indisposition to meddle with varicocele, and we certainly do not meet with many cases in which the inconveniences are very serious. We have laid the gist of M. Delpech's memoir before our surgical brethren, in order that they may put his proposal to the test of their experience, if fit and proper cases should present themselves. By the way we may oberve, that in one of the Parisian hospitals, the spermatic artery has been tied for varicocele, in order to effect the wasting of the testis. We should not expect much from this operation. Castration has also been performed, but on this we need say nothing. We again recommend M. Delpech's proposals and the result of his cases to the attention of our brethen.

### XIV.

### Encysted Abscess of the Brain communicating with the Ventricles.*

Two cases of this description are reported by M. A. Portal, interne of the Hôpital St. Louis.

*Case* 1. A young man, æt. 36, was admitted into the hospital, on the 28th Jan. 1830, with phlegmonous erysipelas of the left fore-arm. He had fractured the bones six weeks previously, and had used the limb too soon. We need not particularize the treatment, suffice it to say that fluctuation became manifest, incisions were performed, and a great quantity of pus discharged from the neighbourhood of the articulation. Recovery was slow, abscesses formed near the elbow and in the axilla, some pulmonary affection supervened, and the wounds did not begin to cicatrize until the month of March. The suppuration now began to diminish, but the patient began to complain of some pains in the head more severe at night, and accompanied with slight shiverings ; a little pyrexia, and slight indigestion. There was now a slight tendency to delirium occasionally, and the suppuration was of serous quality. He was however improving, having merely some pain in the head and stiffness in the affected limb, when he died suddenly in the night of the 13th of April.

*Sectio Cadaveris*. The thorax and abdomen presented nothing remarkable. The membranes of the brain were very pale, the cerebral convolutions flattened, and, as it were, compressed. On raising the cerebral mass there was seen at the inferior and middle part, by the sella turcica, some greenish yellow fluid, and pus escaped from under the tunica arachnoidea. On making sections of the brain, a softened portion was discovered in the white matter, about an inch from the anterior lobe. This softening surrounded a rounded, hard mass, about the size of an egg, which proved to be a cyst filled with pus, capable of being turned out with facility from the middle of the softened mass. The parietes of the cyst were a line and a half in thickness, and in consistence resembled an hypertrophied intestine. The internal surface resembled in appearance a mucous membrane, and presented small cicatrices, injected with numerous vessels. At the back of the tumour was an opening communicating with the lateral ventricle of the same side ; this, as well as the opposite ventricle, was filled with pus, the septum lucidum being in part destroyed. The third and fourth ventricles contained pus also.

*Case* 2. A young man, æt. 30, presented himself at the Charité, on the 9th July, 1828. He had suffered for six weeks from violent pains in the head ; the face was pale, soddened ; the eyes weak, lack-lustrous, almost constantly closed, the conjunctivæ injected. The powers of motion and sensibility were perfect in the limbs, the replies to questions slow, but the intellectual faculties remained undisturbed, the appetite indifferent, the pulse not increased in frequency. Bleeding, sinapisms to the feet, &c. were prescribed by M. Lerminier, and apparently attended with benefit, for the patient was able to walk about the garden and enjoy himself. On the 15th he was suddenly attacked with violent pain in the head, the face soon afterwards became red, the eyes injected, he frothed at the mouth, and in six hours he died.

*Sectio Cadaveris*. The membranes of the brain were injected, especially towards the anterior part of the left ventricle, where the arachnoid and pia mater adhered to the substance of the brain. Beneath this was the cavity of an abscess, occupying a large part of the anterior lobe, and extending to the lateral ventricle into which it had penetrated by an irregular opening. The pus in the interior of the ventricle was mixed with a small quantity of serum. The cerebral substance was softened to the extent of two lines round the abscess, and some of the softened portion floated in the cavity. Below, the brain

* Journal Hebdomadaire, No. 7. Nov. 1830.

was indurated, the corpus striatum rendered more grey and more soft. There was nothing unusual about the thoracic or abdominal viscera.

The reporter conjectures that the suddenness of death was owing, in these cases, to the pus escaping from the abscess into the lateral ventricle. This may or may not be the case, but the facts are of interest in shewing the obscure character of diseases of the brain, and the caution to be exercised in the consideration and treatment of the most apparently trifling cerebral symptoms. We may here introduce a case related by Mr. Adams in the fifth number of the Glasgow Medical Examiner. It differs somewhat from the preceding.

"A.B. æt. 32, a muscular little man, had been subject to head-ache for about eighteen months, but did not apply for medical assistance until about six months previous to his death. His general health at that period was so much impaired, that he was unable to follow after his usual employment. He now rapidly lost ground; became paralytic; had constant convulsive twitchings of the left half of the face, with such difficulty of articulating that he was often unintelligible. He was carried off by general convulsions.

AUTOPSY.—In the substance of the posterior lobe of the right hemisphere, an abscess about the size of a pigeon's egg was discovered; and the inner table of the cranium in contact with the diseased part, was carious. No particular diseased appearance was observed in any other portion of the brain. The thoracic and abdominal viscera did not present any diseased appearance worth noting."

---

### XV.

### CASE OF ULCER OF THE RECTUM CURED BY SARSAPARILLA AND THE BOUGIE.

THIS case is communicated to the pnblic by Mr. Lyle in the third number of the Glasgow Medical Examiner. All who have had the treatment of cases of ulcer of the rectum are aware of their painful and troublesome character, but division of the ulcer generally effects a cure.

On the 9th June, 1829, Mr. L. visited Mr. S. a merchant, æt. 48, robust and active, but with countenance indicative of disease. He complained much of pain in the lower portion of the intestinal canal, chiefly on the left side of the rectum, 2½ inches above the anus, which was contracted; pain permanent and agonizing, especially during and for some time after stool; fæces twisted, and never larger than an earthworm; bowels habitually costive; any attempt to introduce the finger prevented by the violent spasmodic action of the sphincter ani. The disease had first appeared when he was between 18 and 20 years of age. Besides these symptoms he had laboured for 12 years under an inflamed vesicular eruption on the perinæum, scrotum, and groins.

The integuments of the perinæum were ordered to be fomented twice daily, the citrine ointment to be applied at night, and the dec. sars. c. to be drunk daily, with a wine-glassful of a purgative every morning. A tallow bougie about the size of a glyster-pipe was introduced every day, and on the 15th Mr. Lyle succeeded in passing the finger into the gut, and detected an ulcer the size of a shilling exquisitely painful, at the part formerly mentioned. The treatment was persevered in, substituting, on the 20th, a Plummer's pill for the decoction of sarsaparilla. The bougie was increased in size, and on the 25th of July the patient is reported as cured.

---

### XVI.

### MICROSCOPAL RESEARCHES ON THE BLOOD IN DISEASE. By M. DONNÉ.*

ALL the world is aware of the avidity with which microscopical researches have been carried on, in the investiga-

* Journal Hebdomadaire, No. 40. Tome vi.

tion of the intimate structure of the organs, tissues, and fluids of the human body. The blood has been chosen, par excellence, as the subject of experiment. We confess that we have hitherto derived little advantage, little useful knowledge, little amusement even, from the various microscopists. No two have looked through the same glasses, and consequently no two have seen the same things; their observations are as numerous as themselves. Yet, as chroniclers of passing events, we are bound to record any fresh inquiries of this kind, though our private opinion of their utility and value may be far from favourable. M. Donné has been examining with the microscope the composition of the blood in morbid states of the body. We shall state, as briefly as we can, the results at which he has arrived.

In many diseases the form of the globules is altered. In an individual in good health the globules of the blood are perfectly round, transparent in the centre, and of an equal diameter. In a person worn out by long illness, whose organs have undergone perceptible alterations, the globules are less numerous, smaller, deformed, and their general and regular equality is gone. M. Donné asserts the following facts.

1o. In the blood of a woman twenty six years of age, dead of gangrene of the lung, and the body emitting the odour of putrefaction, the globules were small and remarkably deformed.

2o. In the blood of a woman dead of puerperal peritonitis, the globules were less deformed than in the preceding instance, but they could not be clearly made out. The fluid effused into the abdomen presented a few, very deformed, globules.

3o. In the serum of the blood of a woman, who had suffered for some time from disease of the brain, and was bled on account of erysipelas, the globules were very small and few; the blood of the clot did not offer a very regular form.

4o. In the blood of a man who was bled for bilious fever with pneumonia, the globules were fine, and tended to become united together.

5o. Serum of a young girl affected with bilious fever:—globules well marked, not very transparent.

6o. Blood of a woman dead of dropsy;—globules in very small number.

7o. Blood of a woman dead of disease of the liver:—globules tolerable, but disposed to be deformed.

8o. Blood of a young man dead of acute peritonitis, treated by mercurial frictions:—globules very deformed.

9o. Blood of another young man under similar circumstances:—globules not possessing their natural form, and some amongst them very large.

Appended to the foregoing are some statements concerning the existence of animalculæ in animal fluids. We all know of the discovery, or reputed discovery, of animalculæ in the semen by Spallanzani, and many know that a subsequent physiologist has accurately described their motions and habits. M. Donné has ascertained that these animalculæ do not exist in the secretion of the prostate. M. Donné has discovered in perfectly transparent liquid contained in an ovarian cyst, some very small living animalculæ. They appeared composed of a series of rings, and the termination at each extremity was obscure. They swam slowly by bending their bodies alternately to right and left, and they were of so minute a size, as not to be more than one-tenth as large as the spermatic animalculæ. We can only say of these observations, that we hope they may be productive of some utility.

---

### XVII.

### Clinical Lectures by Dr. Perry on Burns and Scalds.*

It cannot be denied that much discrepancy of opinion exists on the treatment of burns and scalds, that much uncertainty obtains in the minds of the less experienced members of the profession, and that much bad practice is

* Glasgow Journ. No. XVI.

the necessary consequence. As an instance of this we may mention a case which we lately witnessed. A child was severely and extensively burned, from his clothes having accidentally caught fire. He was taken to a chemist's and soused in cold water. When brought to the hospital where we received him, he was cold and shivering in his half-burned wet clothing. In spite of warmth and stimuli, the collapse increased, and in a few hours the child was dead. We have seen but too many instances of what we cannot but designate incompetence, in the treatment of these injuries; and, as the majority of mankind do not think for themselves, but shape their conduct by the precepts of others, we shall still be constrained to lament the indiscretions of practitioners, till the rules of practice are settled on a firmer basis and the principles of treatment more generally agreed to. It appears to us that surgeons have erred in looking on burns in too particular a manner. A burn, said they, is a burn, and it ought to be treated with heat or with cold, lotion or ointments, flour or cotton, as their fancies led them to imagine, or experience seemed to dictate. Yet surely all this is highly unphilosophical, and theory has been but the herald to empiricism. A certain quantity of caloric reddens the skin and produces a vesication, a greater quantity kills the cutis and it assumes a white appearance, a still larger amount kills other parts or tissues as well as the cutis, and a slough of greater or less depth is the consequence. Are all these conditions of parts adapted for one treatment, to be remedied by one agent or class of agents? Reasoning would seem to say no, and experience in judicious hands confirms its dictates. For our own parts, we have come to the conclusion that no one method of treatment is exclusively appropriate to all varieties of burn.

The author of the paper in the Glasgow Journal, to which we shall now direct attention, must, from his situation in the infirmary of a manufacturing town like Glasgow, have many opportunities of studying the effects of scalds and burns. We are therefore induced to notice his opinions and record his experience for the benefit of our readers. After criticising Mr. Kentish's views, he makes the following remarks:

"It ought to be remembered, that the part generally injured by heat or fire is the skin; a texture liberally supplied with nerves, blood-vessels and absorbents, and these all defended and supported by the cuticle, which adheres firmly to every part, and derives its nourishment from the capillaries. Under the cuticle terminate the sentient extremities of the nerves, forming part of the reticular coat, and supplying the capillaries, thickly spread upon it, with the power of action. When heat is suddenly applied in an over degree, it easily penetrates the cuticle, preternaturally excites the nerves, and increases the action of the exhalent vessels, which throw out the serous fluid which they circulate so rapidly, that the cuticle which defends the extremities of the nerves, is quickly raised, and forms a blister, and if removed, the nervous papillæ are exposed, and sensation increased. The stimulus of the heat being now withdrawn, the capillary vessels having been over-excited fall into a state of atony or debility, and being unsupported by the cuticle, easily yield and become so dilated, that those which formerly only admitted pure serum now admit red globules, and from their distention, swelling, redness, and increased sensibility is the consequence; still the part is not deprived of life, but capable of being excited to healthy action. Or the degree of heat applied to the part may be so great, that the cutis vera shall be partially, or even wholly destroyed, or so far disorganized that it soon dies, and must be separated from the living parts; a process, which in a healthy state of the constitution, nature sets immediately about, leaving an ulcerating surface, which begins to heal around the edges, if the constitution is good and the treatment has been properly conducted, even before the eschar had been fully separated from the centre. Or, 3dly, The skin and subjacent parts may be so completely disorganized by the first injury, that it becomes black and incinerated, and must be thrown off like any other eschar.

If the burn is extensive, and the shock to the system great, as it generally is in such cases, the power and action of the brain and vascular system, are so much diminished by the sudden exhaustion of the nervous energy, that the blood leaves the surface and extremities, and accumulates in the internal and larger vessels to such a degree, as to impede the action of the heart and lungs, producing dyspnœa, and sometimes an effusion from rupture of small vessels takes place, either upon the brain or spinal marrow; and in children, the congestion or ingorgement of the vessels of the brain, unless attended to, frequently ends in serous effusion upon that organ. I have reason to believe, too, in some instances, where the circulation has become so languid that the pulsation could not be distinctly felt, that the blood had coagulated before death in the heart and larger vessels, by which circumstance, restoration by the exhibition of otherwise sufficient stimuli was prevented.

In other cases, where the apparent injury is not so great, the constitution may be so weak, that it is overpowered by the shock; the pulse is feeble, the extremities cold, the patient shivers, is restless, falls into a state of stupor, and sinks without complaining much of pain; or re-action may take place, accompanied with fever, and much constitutional excitement; on the subsidence of which, the patient may sink; this effect the more readily takes place, if too free recourse is had to the lancet, to subdue the excitement of the system.

It often happens that while portions of the skin and subjacent parts are so severely injured, as to be completely disorganized, the surrounding parts may have been only partially over-excited, and still having a portion of vitality, are capable of being acted upon by external stimuli, and consequently, in a state requiring a different topical application from the former; this is a point too frequently neglected in the treatment."

We conceive that, with much that is undeniable, there is some little speculation in the foregoing quotation. On the withdrawal of the stimulus of heat, says Dr. Perry, the cutaneous vessels become debilitated, yield, and carry red blood instead of serum. Now a scald produces redness of the skin, which remains for some time. If the skin in this state be stimulated we frequently have vesications and the other consequences of inflammatory action: but if it be soothed by sedative applications the redness, heat, &c. for the most part pass away quietly enough. Can it be said that the capillaries were dilated and debilitated? if they were so, that debility was aggravated by stimulants, relieved by sedatives. Dr. Perry relates five cases, in order to shew the description of treatment applicable to the different varieties of burn.

*Case* 1. "A. B., a child about two years of age, was scalded over the face, neck and breast, with hot or boiling water; the cuticle was in various parts raised into blisters, but not abraded, except a part on the left side of the breast where the skin had been rubbed off in removing the clothes, the whole face and neck were of a high red colour; cold water had been applied in the first instance; twenty minutes had elapsed from the time of the accident, when equal parts of whisky and the common vinegar of the shops, gently heated, were applied with linen cloths, which were kept constantly wetted, without their being removed. Carded cotton was at the same time applied to the breast, the child soon ceased crying, and in less than half an hour fell asleep. At the end of three hours the wet cloths were taken off and the parts examined; the blisters had fallen, and the redness was nearly gone; the cloths were again applied, wetted with the spirits as before, with a less proportion of the vinegar, and continued till next morning; the child passed a good night; the blisters had disappeared; the face had rather a pale bleached appearance, the cloths were allowed to dry, then removed, and a little of the resinous ointment, to be afterwards described, was smeared over the face, and that portion of the neck to which the cotton was not applied. In three days after, the patient was allowed to have his face wholly uncovered. Slight desquamation took place over some parts of the face, particularly the left cheek, and under the chin. The cotton remained adherent for about twelve days; when removed, the cuticle had separated, leaving the parts underneath sound, but of a reddish colour, and the redness of the face had wholly disappeared. There was no constitutional treatment except a laxative required."

In the next case in which the face was scorched by gun-powder, being swelled, and in parts vesicated, warm vinegar and spirits were applied to one part, and cotton-wool to another. In twenty-four hours the spirituous wash was discontinued and a turpentine ointment applied. The parts thus treated were well in six days, the cotton separated leaving the surface beneath it well in twelve days. The next case will shew the treatment of a burn of more severity.

*Case* 3. "J. B., a collier. 2d August, 1831.—This morning about three o'clock, while at work in a pit, with his face, neck and arms uncovered, was exposed to an explosion of inflammable gas, and admitted into the infirmary about seven o'clock, a. m. Both arms, hands, and face, with a part of right side are vesicated, and of a dark ashy colour, except where the vesicles are broken, where the skin beneath is of a brownish colour. The neck and ears are also scorched. Is chilly, with some nausea. Bowels costive. Had forty drops of laudanum on admission.

℞. Sub. mur. hydr. gr. vi.
Opii. gr. i. m. sumat. statim.

℞. Terebinth. Venetæ, oz. i.

Adep. suill. oz. iii. m. ft.*

Liniment. hoc curent. brachia et manus.

The cotton-wool to be applied to the ears and neck.

Next day the face and arms were much swollen; tongue dry, with much thirst. Pulse 100. No stool. *Habt. statim bolum commun. et enem. domest. Vesp. repet. mist. diaphoret.*

On the fourth day after admission, on changing the dressings, the arms and side were found to be suppurating freely, and less swelled. The bowels had been freely opened. Tongue dry; much thirst; pulse 100; sharp. *To have a solution of salts with senna, and the diaphoretic mixture continued.*

9th. Has improved considerably; heat of skin moderate; pulse 100. Expresses some desire for food. Tongue white; thirst continues. Complains of smarting pain from the ointment. *Ointment to be diluted with equal parts of hog's lard. To have* ℔i. *of porter daily. Cont. alia.*

10th. Was put upon full diet, and the same treatment continued, under which he began to improve rapidly. The cotton separated from the ears and neck, about the sixteenth day after admission, leaving the parts beneath sound. The fingers were longest in healing, having been most severely scorched. They continued to be dressed with the diluted terebinthine ointment till the 10th September, when he was dismissed cured, the skin everywhere smooth, and without scars."

In the foregoing case it will be observed that the extent to which the tissues were altered and injured by the heat, is not accurately described. This is a fatal omission. We need not detail any other cases. They are mostly specimens of burns of considerable extent, which were treated by terebinthinate applications and proved fatal. In one case, that of a female aged 45, the burn was extensive on the left side of

* "The above ointment is best mixed by a spatula, upon a marble slab, when it forms a soft liniment, and can be applied without removing the dressings, which ought to be changed as seldom as possible. In cases where the disorganization does not go deeper than the cuticle, this ointment requires to be mixed with an equal part of hog's lard, or, if necessary, a little olive oil. Whenever its application begins to give pain, it ought to be diluted."

the chest, and the part was hard and of a dark colour. Under the emeto-cathartic mixture at first, with turpentine dressings, and subsequently tonics and generous living, the slough separated, and the patient was dismissed cured in two months.

Dr. Perry started in the early part of his lecture or paper with condemning exclusive and empirical practice, yet, so far as we see, his own is exclusive, if not empirical. He seems to have one notion—that the parts are debilitated; one creed of treatment—that they ought to be stimulated. Now we do not believe that skin which is merely reddened or vesicated, is to be treated as skin destroyed, and to be thrown off. Is not skin merely vesicated by heat, under nearly the same circumstances as skin vesicated by cantharides? The action is more rapid, but in kind it is similar, in consequences it is analogous. Well, we treat a blistered surface by mild applications, whilst we are directed to treat a burn'd one by stimulants. It may be said that the reasoning is specious, but not borne out by the results of practice. We contend that it is so. We have treated many burns of all kinds and degrees, and we do prefer the sedative treatment for those of the kind we have mentioned. If a patient applies to us with vesication and extensive redness of an extremity, or even of the trunk, *the cutis itself not being killed* partially or totally, we wash the surface with tepid or even cool goulard water, and dress it with lint or linen spread with a mixture of the ointments of zinc and lead, taking care to prick with a needle the larger vesications. Over the whole we lightly apply a roller, and if necessary keep it wet with goulard water. This dressing is to be continued for two or three days, when the redness will usually have disappeared, and the vesicated skin have separated, leaving the exposed cutis either suppurating or cicatrized. If the latter, of course little more need be done; if the former we have recourse to the following means. We wash the exposed surface, which looks somewhat like a strawberry from the prominent red points in the cutis, with a strong solution of sulphate of zinc, gently dry it, and then dust it freely with the lapis calaminaris or some other absorbent powder, applying over all some ointment spread on linen. Frequently this will effect the cicatrization of an extensive surface, but if portions still appear inclined to suppurate we repeat the application of the powder as often as may be necessary. We can conscientiously assert that plan of treatment, conjoined with the proper use of purgatives, salines, and, when necessary, tonics, is more successful than any other which it has been our lot to witness. There are many particulars of course into which our narrow limits prevent us from entering.

When the cutis is killed we are decided advocates for stimulant applications, and we know of none which are preferable to turpentine. Cotton has lately been cried up; we have seen it tried in some cases, but with no great success, and on the whole we think it not generally applicable. The following opinion of Dr. Perry, emanating from a place where cotton was said to be so successful, deserves consideration.

"In all cases, where no good opportunities exist, of the patient being carefully dressed, and attended to, it is an exceedingly good application, particularly in scalds and superficial burns. Where the burn is so severe, that subsequent ulceration must take place, the application of the cotton wool is sometimes attended with very unpleasant circumstances, particularly in summer; of which the breeding and nestling of maggots in the purulent discharge, and about the body of the patient, producing excessive irritation, is not the least."

In mild cases we imagine that the treatment which we have described will be found more convenient and successful; in severe ones the objections raised by Dr. Perry are of a serious character. On the whole we agree with Dr. Perry in his reprehension of the cold water method of treatment, but we think he has conjured up more local debility than really exists, and attempted to lay his fancied sprite with a stimulating spell, more potent than is absolutely necessary in all cases. We may return to this subject on another occasion.

---

XVIII.

A TREATISE ON THE DISEASES OF THE HEART AND GREAT VESSELS, COMPRISING A NEW VIEW OF THE PHYSIOLOGY OF THE HEART'S ACTION, ACCORDING TO WHICH THE PHYSICAL SIGNS ARE EXPLAINED. By J. HOPE, M. D. &c. &c. &c. Octavo. London, 1832.

IT is now but a few years since Laennec's discovery was bruited in this country, since the Editor of this Journal had the good fortune to be the first that promulgated in Britain his ideas and experiments. The party that opposed all novelty in all things was powerful then; wisdom was only with their ancestors, and by descent with themselves; what was not known formerly could not be discovered now; the eye and the hand and the nose were to be employed in the diagnosis of disease, but the ear was not: and, in fine, we were to make no improvement, because hitherto little improvement had been made. The party we mention was powerful then, and we will not say that it was descended from that holy band, which had made the starry Galileo too familiar with his woes. This party naturally opposed the stethoscope and the stethoscopists; the first was an "inutile lignum," the last were a credulous crew. And what were their arguments? We will not reiterate them now, we would not, for very charity, call up such frailties from their dread abode. The battle has been fought, and with whom is the victory? Alas! for the children and advocates of things that were, the inutile lignum has grown into a noble tree, and its branches have spread and its twigs have shot into the recesses of their own high places. The no-stethoscope cry is heard no longer; those who raised it are ashamed of it, those who listened to it laugh at it. Men of sense and of candour have examined for themselves, they have tested the merits and defects of auscultation where they should be tested, in the hospital or sick-chamber, not in the *persiflage* of conversation, or the formal disputes of a debating society. Of those who have resorted to the enquiry with this spirit and in this manner, very few have condemned auscultation as a system of fallacy or delusion, though some have found their patience inadequate to carry them through the investigation. The opponents are silenced, the advocates consider it necessary to exert themselves no longer, and those who see with the eyes of 1831, that is to say, whose minds are not cast in the mould of some centuries ago, may perceive the more aspiring, intelligent, and active of the rising generation of professional men, engaged in the study of auscultation, as a component part of their medical education. There is nothing surprising in this to him who has studied, even in a careless mood, the history of his species. We cling in age to the tales of our childhood, to the knowledge obtained by the vigour of our manhood, and time which lays the sophist and the sage in the dust, consecrates too often both their folly and their wisdom.

It is not necessary now to preface an article on auscultation, by proving that such a study is not absolute nonsense. It has been decided by the voice of the profession, that it must in future make a part of a scientific physician's and surgeon's education. But this science, or department of science, is not perfect, (we should wonder if it was so,) and the labours of those who pursue the investigation must be directed to the confirmation of some points, the correction and the elucidation of others.

We hail the appearance of the work, the title of which we have placed at the head of this article with a high degree of satisfaction. It is not the hasty speculation of a day, got up to meet a passing ferment, engendered in imperfection to be entombed in oblivion. We have reason to know that Dr. Hope has devoted some time and attention to the study of the subject on which he writes, and although we have no doubt that he is better read than to be unacquainted with what others have thought and said, yet we feel equally confident that his work is not a mere compilation, a dictionary of quotations, octavo-rigged, a privateer fitted out by merchants of the Row, sailing under false colours to capture the weak and inadvertent. Before this review is completed we shall enable our readers to determine whether our good opinion is unfounded.

The article on cholera in this number of our Journal has curtailed our space. We shall therefore be unable to give a complete account of the work of Dr. Hope at the present moment, in fact, we shall merely be enabled to commence an analytical review, which will probably be completed in our next.

The introduction to the volume, occupying thirty pages, is deserving of attentive perusal, as it points out the scope and aim of the author, and marks in a satisfactory manner the deficiencies and defects of the recognized theories and opinions on the affections of the heart. Dr. Hope properly argues, that dangerous and intractable as these diseases unquestionably are, they may yet be reasonably expected to be rendered less so, when careful clinical observation of the general and physical symbols of its condition presented by the heart, shall enable the physician to detect the earlier variations from natural structure, the less prominent alterations of natural function.

"Many think that the expectation of effecting an improvement in the diseases of the heart chimerical: and they think so because, not being accustomed to recognise the diseases in question before they have attained an advanced stage, they are pre-occupied with the old and popular idea of their incurability. To such it might, perhaps, be a sufficiently philosophical answer to reply, that an improved knowledge of the nature and causes of a disease, must alone necessarily lead to an improvement in the treatment; and that therapeutic weapons are dangerous when wielded in the dark. But here we may go much farther: we may say that, by the improved means of diagnosis, the maladies under consideration may be recognised, not only in their advanced, but in their incipient stages, and even when so slight as to constitute little more than a tendency. We may say on the grounds of incontestible experience, that, in their early stages, they are, in a large proportion of instances, susceptible of a perfect cure; and that, when not, they may, in general, be so far counteracted as not materially, and sometimes not at all, to curtail the existence of the patient. We may accordingly predict, that the term 'disease of the heart,' which at present sounds like a death-knell when whispered by the physician, will hereafter become by familiarity not more alarming than the term *asthma*, under which it is frequently disguised."

Such are the *direct* practical improvements to be expected from a better knowledge of the diseases of the heart. But there are others of equal magnitude.

"It has been stated by M. Richerand, repeated by Bertin, and echoed by all who are conversant with this class of maladies, that 'hypertrophic enlargement of the heart is more closely allied to apoplexy and palsy than the apoplectic constitution itself.'*

Should the hypertrophy be recognised, its effects on the brain may be counteracted by judicious treatment: should it be overlooked, the patient, with a view to reducing his *apoplectic fulness of habit*, is ordered smart exercise, which by increasing the action of the heart, already too powerful, causes a preternatural determination of blood to the brain and induces the apoplectic or paralytic seizure. According to evidence hereafter to be adduced, the majority of those who are cut off prematurely in the apparent enjoyment of good health, sink under the circumstances described.

Again, there are few more common

---

* "This constitution consists, according to the proper idea, in a broad, robust frame, full habit, and florid complexion. It is in general attended with an unusual size and thickness of the heart."

and certain exciting causes of palpitation and difficulty of breathing in disease of the heart, than derangement of the stomach. What happens to the patient in this case? Tracing the attack, in perhaps every instance, to dyspeptic fit, he naturally concludes that the latter is the cause: that it is 'all indigestion.' 'Good air and plenty of exercise' are the remedies recommended: the result is an apoplectic seizure. The circumstance that before the introduction of the new mode of exploring diseases of the heart, they could rarely be detected in their early stages, contributed to the error in question. For, as patients frequently recover from the early stages, the recovery was regarded by those who assumed this class of diseases to be incurable, as a proof that the affection was merely dyspeptic. Hence dyspepsia acquired the reputation of producing certain symptoms, particularly in the head, which are in reality foreign to it, being exclusively the results of a co-existent disease of the heart.

There prevails another error, the converse of the above—that of mistaking the nervous or really dyspeptic palpitation, for disease of the heart. The frequency of cases of this kind, especially amongst men of studious habits, (and more particularly, I have noticed, among those of my own profession,) is truly surprising; and, as it has always been considered difficult, and by many impossible, to distinguish the two affections, the alarm created is sometimes distressing. Having thought this subject of so much importance as to demand a separate article, I shall here only say, that so far as my own experience enables me to judge, the distinction may be made with ease and certainty.

An immense proportion of asthmas —and of the most dangerous and distressing cases, result from disease of the heart: the same may be said of dropsies, especially those that are universal. If the cause is overlooked, the asthmatic is harassed with a farrago of inappropriate and unavailing, not to say pernicious remedies; and the hydropic is treated with dangerous activity; or for imaginary affections of the liver, the lungs, or the kidneys. On the other hand, if the cause be detected in the incipient stage, by precautionary measures, both the one effect and the other may in general be prevented.

In acute rheumatism, there is no more common and formidable source of danger than inflammation of the heart and its investing membranes. Should it be overlooked when existing in a severe form, (and even then it is one cf the most obscure and insidious of maladies,) the patient almost invariably dies from the immediate effects of the attack, or becomes a short lived martyr to an incurable organic disease of the heart.

There is scarcely a disease of the heart, accompanied with obstruction of the circulation for any considerable period, which is not productive of enlargement of the liver, and, sooner or later, of its ordinary consequence, abdominal dropsy. Yet there are few common facts in medical science less generally known than this intimate connexion between the heart and the liver. The dropsy is ascribed to the latter; the treatment extends not beyond this organ; the unknown cause continues to reproduce its effect, and the patient, if he obtain relief at all, only obtains it to undergo a speedy relapse.

Individuals affected with disease of the heart are peculiarly liable to inflammation of the lungs; and such inflammation, as I have endeavoured strongly to inculcate throughout this volume, is singularly rapid and destructive. Yet if, from ignorance of the state of the heart, free depletion be practised on the ordinary principles, the patient may sink suddenly after the first or second abstraction of blood. I have more than once witnessed this catastrophe, and few practitioners of experience have not seen the same.

In fever and inflammation in general, disease of the heart may impart to the pulse dangerously deceptive characters of hardness, fulness, weakness, or irregularity, and the patient may be bled too much from the prevalence of the former characters, or too little from the presence of the latter.

Thus it is seen that the practical improvements to be derived from a better knowledge of the diseases of the heart, extend, not to the diseases of this organ alone, but to a multitude of the most formidable maladies incident to the human frame. There is, in short, scarcely an affection with which disease of the heart may not be more or less interwoven; and, 'if,' to use the language of Senac, 'we would not pronounce rashly on an infinity of cases; if we would not harass our patients by noxious and unavailing remedies; if we would not accelerate death by treating certain diseases like others which are entirely different; nor be exposed to the disgrace of seeing our diagnosis falsified by the results of dissection; finally, if we would not have danger to be imminent, whilst we are under the blind impression that it is remote, we must study the diseases of the heart.'"

In the first chapter Dr. Hope very properly considers the relative anatomy, or the anatomy of situation, of the heart, and the application of percussion to the diagnosis of its conditions. The following important passages cannot be materially condensed, and should not be omitted.

"As the apex and body of the heart are free, while the base, secured by the great vessels, is comparatively, though not absolutely fixed, the organ turns in a slight degree upon its base with each alternate movement of the diaphragm, the descent of the muscle causing its longitudinal axis to assume a more vertical position, and the ascent throwing it transversely to the left. It is necessary, therefore, that the auscultator fix upon some given point at the base, which may serve as a mark and guide for his exploration of the situation of the organ. The point which to myself has appeared the most certain, is, the pulmonary artery. This vessel, midway between its origin and the place where it divaricates into the two trunks distributed to the lungs, bulges at the interspace between the second and third left ribs close to the sternum, a circumstance which, as well as the situation of the other parts of the heart, I have carefully ascertained by forcing needles through the thoracic walls, at given points, into the viscera beneath. The situation of the pulmonary artery was, also, well displayed by the dilatation of that vessel described in case xx. (Weatherly.) A line drawn from the inferior margins of the third ribs across the sternum, passes over the pulmonic valves a little to the left of the mesial line, and those of the aorta are almost directly behind them. From this point the aorta and pulmonary artery ascend; the former inclining slightly to the right, coming in contact with the sternum when it emerges from beneath the pulmonary artery, and following, or perhaps rather exceeding, the mesial line, till it forms its arch; the latter, which is, from the first, in contact with the sternum, inclining more considerably to the left, until it arrives at the interspace between the second and third ribs above described. A vertical line coinciding with the left margin of the sternum, has about one third of the heart, consisting of the upper portion of the right ventricle, on its right; and two thirds, composed of the lower portion of the right ventricle and the whole of the left, on its left. The apex beats between the cartilages of the fifth and sixth left ribs, at a point about two inches below the nipple and one inch on its sternal side.

The lungs descend along the margins of the sternum about two inches apart, and overlap the base of the heart, slightly on the right side, and more extensively on the left: then, receding from each other, they leave a considerable portion of the right ventricle, and a less extent of the lower part of the left, in immediate contact with the sternum.

The right auricle is in front of the heart, at its right side and upper part. One portion of it is overlapped by the right lung, and another, principally the appendix, is in contact with the sternum. The left auricle is situated deep behind and to the left of the heart at its upper part, opposite to the interval between the cartilages of the third and fourth ribs. The extremity of the appendix is visible in front, but, when the volume of the heart is natural, it is not in contact with the sternum, being considerably overlapped by the left lung. The pericardium ascends on the great vessels as high as the commencement of the arch of the aorta, and opposite to the second ribs.

When the heart is enlarged, its longitudinal axis becomes placed more transversely, and its lateral diameter is increased. Hence, the right ventricle projects more considerably to the right, sometimes under the whole breadth of the sternum; and the left extends far beyond its usual limits to the left, sometimes elevating by compression that portion of the lung which overlaps it, so as to bring nearly its whole surface, and the tip of the auricular appendix, in contact with the sternum. In addition to being broader and placed more transversely, the organ descends lower than natural—its apex sometimes beating between the sixth and seventh ribs, and its pulsation extending to the epigastrium.

When the right auricle is dilated or gorged, it extends upwards and to the right, and comes more extensively in contact with the sternum.

When the pericardium is distended to the utmost with fluid, it forms a pear-shaped bag, the top or narrow extremity of which sometimes mounts even above the second rib: its sides are nearly in contact with the sides of the heart, while its front is separated from the anterior surface of the heart, in the dead subject, by two or three inches of interposed fluid.

From the above description the auscultator will understand in what situations to explore the lesions of the various parts of the heart."

Dr. Hope mentions a few particulars on the mode of employing percussion, to which we may allude. When great delicacy is required, percussion on the *plessimeter*, lined with wash leather, is preferable to that on the back of the fingers. Of course, percussion on a solid body emits a dull sound, but on a body containing air, elicits a hollow one. M. Piorry has also discovered that if a solid body be placed beneath a sonorous one, the sound is modified in consequence, and is intermediate between hollow and dead. This ensues from the vibrations of the air in the sonorous body, being checked by impinging on the more solid substance.

We regret that we must defer until our next number the continuation and conclusion of our account of the present work. The lateness of its publication, and the all-engrossing malady which now occupies men's minds, and threatens to occupy their bodies, must plead our excuse with the talented and indefatigable author, as well as with the public.

---

### XIX.

[*Appendix to Article on Cholera.*]

### Cholera in England.

When this mysterious epidemic was advancing westward from Moscow and St. Petersburg towards the shores of the Baltic and North-Sea, the panic in this country was great—when it reached Hamburgh, it was still greater—but when it burst on our shores, and was announced to be an *imported* disease, the consternation was at its height. So long ago as last June, the Editor of this Journal hazarded a prophecy (a hazard which he was a fool to run) that, should the epidemic cholera invade Great Britain, it would be greatly modified in its character, and much abated (comparatively speaking) in its fatality and rapid dissemination, by the nature of our climate and the manners and habits of our population. This prophecy was immediately met by other prophecies, promulgated through the same channels, intimating that, from the facilities of our communications, and various other circumstances, the cholera, when once *introduced* on British soil, would spread like wild-fire, and produce havoc the most prodigious. The final fulfilment of these prophecies is yet in the womb of time; but as far as the events have yet gone (10th of December) the Editor has but little cause to repent of his prognostications. Without going, at present, into the question of the exact period at which the epidemic made its first appearance in this country, we shall take for granted, the dicta of the Board of Health, which dates the first death on the 26th of October last. On the 10th of December, or nearly seven weeks after its outbreak, what was the extent of its *travels*—for this is the fashionable term for its propagation? We are rather at a loss for a simile or example to illustrate the journeys of this scourge, which was to go much faster than a steam-carriage. It crept, like a skulking hyena, from one dirty lane to another, seizing chiefly on those who were half in the grave already, or who, from terror, were flying before the enemy. It rarely or never attacked those who boldly faced the foe.

During the above period, the disease confined itself to Sunderland—and to the very worst parts of Sunderland—localities which might well compete, in filth, poverty, and wretchedness, with the back lanes of Fondi and Itri—that sink of squalid sordidness, the Jews' quarter on the banks of the Tiber—or the dunghill dens of the Cretins in Sion or Martigny! The scourge that was to spread like wildfire, or dart with the rapidity of lightning from city to city, and from town to town in Great Britain, wasted nearly seven weeks, in carrying off about 120 of the most decrepid, drunken, famished, filthy, and aged inhabitants of a sea-port town! We need not, of course, conclude that Newcastle or any other great town can finally escape an epidemic, whose mysterious cause has pervaded a great part of the Old World, and will probably reach the New.*

But it is very curious to observe the change of tone in exclusive contagionists, even in the course of a few months. An ingenious friend of ours has constructed an instrument for measuring the temperature of the contagionists. He calls it a LOIMOMETER, or pest-gage, the highest degree of which is marked ULTRA-CONTAGION—the middle is CONTINGENT CONTAGION—and the lowest is NON-CONTAGION. It is very evident, from the following official records, that the ultra-contagion temperature has fallen surprisingly in less than a single month.

State of the LOIMOMETER at the Board of Health, 20th October, 1831.
[120° or PLAGUE TEMPERATURE of Egypt.]

" Immediate separation of the sick from the healthy"—" conspicuous marks on infected houses"—" rags, papers, old clothes, and hangings to be burnt" —" dead to be buried in the vicinity of the house selected for cholera patients" —" all persons employed about the sick (including the doctors, of course) to live apart from the rest of the community"—" all articles of food to be placed in the front of infected houses, and received by one of the family, *after the person delivering them shall have retired*"—" convalescents and *all those who have had communication with them*, to be kept under observation for a period of not less than 20 days"—" all intercourse with an infected town and the neighbouring country to be prevented"—" troops, or a strong body of police to be drawn around infected places, so as utterly to exclude the inhabitants from all intercourse with the country, if so fatal a disease should ever shew itself in this country," &c. &c.

State of the LOIMOMETER at the Board of Health, 14th November, 1831.
[92° or low fever heat in England.]

" Cholera is not more infectious than fever"—" all measures of coercion for the prevention of intercourse with infected persons or places to be deprecated; when tried on the Continent they have invariably been productive of evil"—" this disease *seldom* spreads in families, and *rarely* passes to those about the sick, under proper observances of

* Since writing the above, indeed, the disease has commenced, or rather *re-commenced* in Newcastle—and the wonder is that it did not spread thither and to many other places long before. The report from Sunderland this day, 12th December, is 17 new cases, 4 deaths, and 23 recoveries. If the epidemic assume no more formidable character than this, England need not despond!

cleanliness and ventilation." "It will *not*, therefore, be necessary, when there is space, and where due attention is paid to cleanliness and purity of air, *to separate members of families actually affected by the disease*—nor to insulate individual houses, *unless in cases of crowded, filthy, badly-ventilated habitations, and other contingencies, which involve the health of all.*"

We entreat the attention of our brethren to these two sanitary documents, issued within *twenty-one days* of each other, from the highest authorities in the land, and say whether the Loimometer of the high-contagion party did not fall, in that short space of time, from plague-heat to that of "CONTINGENT CONTAGION." The Central Board tells us, in fact, that, to take away filth and foul air, is to take away the power of cholera to propagate itself by contagion. Is not this the very doctrine which the Editor of this Journal maintained throughout the whole period of the panic. In the TIMES JOURNAL, of June the 27th, he maintained this doctrine—in the same journal of October the 29th, before the Central Board was established, he thus expressed himself, —"That a focus of *infection* may be generated *occasionally*, in deep cellars and the crowded hovels of poverty, I do not doubt. The same thing takes place every year, *in fevers and other diseases.*" Now we ask the profession—we ask the ultra-contagionists themselves, what more is contained in the Curriculum of the Central Board, than is contained in the foregoing short passage?—Yet the Editor is taunted with having "changed his opinions"—with having advanced on the Loimometer from non-contagion to "contingent contagion"—when the foregoing *facts* prove, beyond the possibility of denial, that the ultra-contagionists have dropped, in twenty-one days, from the plague-temperature, down to the very point which the Editor fixed on as his creed, when the ultra-contagionists were in the plenitude of their power—though, as it soon after appeared, on the brink of their fall!

The Contingent Board (by which we mean the Central Board) tells us that the epidemic cholera is not more *infectious* than the ordinary typhous fevers of this country. However we may respect, individually, the members of this, or of any other Board of Health, we have lived long enough in the world to know that a BOARD of HEALTH is not infallible in its doctrines or conclusions. Were they infallible, we should not have to record upon our pages, the most extraordinary change of doctrine, in three short weeks, that ever was recorded in the annals of medicine! But we will assume that the dicta of the Central Board are the dicta of Omniscience—that no possible error can creep into their conclusions—and that their fiat is fate itself. The *infection* of cholera is *not* more powerful than that of the typhus of our own country. Well. Derobe a typhous patient of *filth and foul air*, the contingencies to which we have alluded, and you take from the fever the power of propagation. You may then feel his pulse, examine his tongue, analyse his evacuations, press his abdomen—and, when he dies, dissect his body, with about as much chance of catching the fever, as of learning from him the secret of alchemy. The infection, then, is a contingency. Take away that contingency and you destroy the infection. By what species of logic are we to maintain that this *contingent infection*, the creature of circumstances, is an essential and inseparable character of the disease—seeing that we can produce it by crowding and filth, and annihilate it by ventilation and cleanliness? Let us compare typhus or cholera with a really and an essentially contagious disease—namely, small-pox. Place a patient with small-pox in a ward of 500 feet in length, by 50 in breadth. Let every possible ventilation and cleanliness be enforced—and then bring an uninoculated or unvaccinated child into *contact* with the variolous subject. Would the contagionist do this with his unprotected child? Not he! But he might bring his child into contact with a typhus patient circumstanced as above, with the most perfect security. Yet this typhus is the standard or measure, which Drs. Russell and Barry have given us, whereby we are to estimate the infectious power of cholera.

We are told, by men who have never seen the epidemic cholera in this or in any other country, that the disease may be discriminated, even by a child, from any grade of sporadic cholera—even by the smell. Yet in the case of Jordan, who died in Newcastle, towards the end of November, Dr. Daun could not say whether it was the Asiatic or the indigenous cholera of this country! The details of the case were submitted to the Central Board of Health in London —and they could not decide! They could only say, in imitation of the Delphic Oracle of Apollo, that it was—"suspicious"!! This man, Jordan, died in the same locality where a man of the name of Reay died two or three months previously, with the same symptoms—namely, in the dirtiest part of Newcastle. The authorities on the spot disclaim all idea of communication between Jordan and any person in Sunderland. But it comes out that this man was very dirty in his habits—in his vocations—and that, in addition to all this, he kept a PIGGERY, in which he spent a great part of his time! According to the testimony of his wife, he was subject to what Sydenham calls "griping of the guts"—but having died one day, the Mayor of Newcastle shewed his zeal, by reporting his death to the Government of Great Britain! The coals of Newcastle, as a matter of course, were subjected to 10 days quarantine in Sandgate Creek, while free communication was allowed between the house and inmates of the deceased, with all England! So much for the consistency of the contagionists!

In respect to the *introduction* of cholera into Sunderland, the contagionists of our medical societies, and of our anonymous writers in the periodical press, consider it as a matter demonstrated, that some healthy sailors brought it from Hamburgh, in a return collier. The King, however, who is no bad authority upon this occasion, tells us, in his speech from the throne, on the 6th of December, that, "whether it (the cholera of Sunderland) is indigenous, or has been imported from abroad, is a question involved in much uncertainty." This is not kind in his Majesty, to throw the shadow of a doubt upon a question which had been settled by a few of his liege subjects in various parts of the kingdom (always excepting Sunderland, where of course, they could know nothing about the matter) long before the speech issued from the throne! If then Jordan's case was one of *sporadic* cholera, it shows the difficulty, if not impossibility, of discriminating it from one of the epidemic, or as it is now called, the Asiatic disease. If it was the real Continental cholera, how is it that it did not immediately spread in the same neighbourhood? This, at least is a consolatory reflection.

In this place we cannot refrain from noticing an important discovery, or rather the confirmation of a discovery, made at Sunderland, and promulgated widely. We have all heard much about cholera travelling from one country to another; but it was reserved for the writer at Sunderland to give us a well-marked case of "A TRAVELLING CHOLERA." A woman travelled from Sunderland to Houghton, a distance of six or seven miles. She was seized with cholera the same day, and died at Houghton. This is a case of TRAVELLING CHOLERA! The sagacious observer designates it as though he suspected no particular relationship between the woman and the cholera. No. It was by mere accident that they travelled on the same road—by mere accident that they travelled on the same day—by mere accident that this disembodied evil spirit took possession of his fellow-traveller at Houghton, and put a period to her existence. If this be the way in which cholera *travels*, why then ague travels thus every year, from the fens of Lincolnshire to the green hills of Erin. And verily we suspect that the reporter has produced a very awkward illustration of the mode in which cholera *travels* from place to place. We believe that many a person has carried the disease itself, in a state of incubation, from one point to another, when it was supposed that the *contagion* was wafted by the air or by fomites from place to place. Considering the emigration that must have taken place from Sunderland to the neighbouring towns, it is astonishing that great numbers should not have travelled with the seeds of the disease in them, and exhibited it far beyond the confines of Sunderland! And here we cannot help taking notice of a watch-word by the ultra-contagionists — namely, the necessity for PRECAUTION. They thereby insinuate, we should suppose, that the opposite party, according to their tenets, inculcate *no precaution*. But let us ask what they mean by PRECAUTION? Do they mean the revival of the pest-laws, as recommended by the Board of Health in October last, and in the Quarterly Review? or do they mean ventilation and cleanliness? If the latter be their meaning, the anti-contagionists and the contingent contagionists enforce, as the very foundation of their doctrine, the removal of filth and foul air. What, then, we again ask, is there in the "*precautions*" of the contagionists, which is not contained in those of the anti-contagionists—except the pest-regulations, which even the contagionists themselves have renounced? If, then, the sanitary precautions be precisely the same, whether prescribed by one sect or the other, let us hear no more of those accusations thrown out upon each other, respecting the sins of omission or commission, on the subject of hygiene.

If we prognosticated, many months ago, that cholera, if it came to this country, would come in a mitigated form, we have the assertion of the King —Regem quis audeat dicere falsum— that our prognostication has not been entirely without some foundation in truth. "Its progress (says His Majesty) has neither been so extensive nor so fatal as on the Continent." When we consider the *weak point* which has been first invaded by the enemy—when we consider that the invasion took place, at the very latest, in the month of October—when we find that cholera spent seven weeks in what may be called the suburbs of Sunderland, carrying off debilitated, aged, and diseased victims, scarcely assailing a single individual in the better classes of society—when we reflect that free intercourse enabled it not, in that time, to get beyond the first spot which it fixed on—have we not some reason to hope and to expect, that the ravages of the disease will not be greater or more rapid when it invades other towns and places of England? Is it not prudent to indulge this hope and this expectation, even if our anticipations should turn out to be unfounded? Terror predisposes the timid more to the assaults of this epidemic than all the gin and porter which they drink—the panic of Sunderland has deprived our poor of coals—has put our ships in quarantine, in every port from the North to the South Pole—will cost our merchants some millions of money—will cripple our commerce for years—and by the distress and poverty thus induced, will destroy more, perhaps, by want and chagrin, than the disease itself would by its actual presence! Such are the fruits of unnecessary, unmanly alarm. Unnecessary alarm, because cholera is acknowledged by the highest authority, to be not more infectious than fever—unmanly alarm, because it is contrary to all precepts, ancient and modern.

Rebus angustis animosus atque
Fortis appare.

Here we would beg to draw the attention of our brethren to a very important subject—the medical constitution of the year 1831 in this country. We believe it is a fact established beyond all doubt, that, ever since the month of May last, a disposition to gastric irritation and bowel-complaints has obtained, in a degree infinitely exceeding that of any former year for a considerable period. This gastro-intestinal irritability has not been confined to the lower classes of society, but has prevailed among all classes. For our own parts, we can safely aver that we have seen more cases, even in the better ranks of society, of diarrhœa, dysentery, and cholera, during the last six months, than during the preceding twelve years. Numerous instances have come under our observation, where people could previously indulge, with impunity, in irregular habits of the table; but who now paid dearly for any such latitude in food, especially in fruits and other kinds of vegetables. The public journals and the experience of our contemporary brethren, as well as our own, have furnished numerous examples of severe and even of fatal cholera. Dr. Marshall, Dr. Brown, Dr. Fergusson, Dr. Burne, and many others, have given their testimony to this effect. We refer, in particular, to the excellent letter of Dr. Brown, of Sunderland, published in our EXTRA-LIMITES, for proof that cholera prevailed in Sunderland, and in all the neighbouring districts, to a very great degree, and often fatal in its terminations, ever since July last. It has been the custom to draw vivid pictures of extreme cases, and hold them up to public view as specimens of the Asiatic cholera in this country, while the various minor grades and forms of the disease were slurred over. The "malignant cholera" was held forth as THE cholera, while diarrhœa and common cholera, far more numerous and far less fatal, were, for a time at least, kept under distinct heads. Let us compare this procedure with epidemics of other diseases. Suppose we were to employ the force of our pens and our imaginations in portraying extreme cases of variola, delineating, and correctly too, the whole body of a fine boy or girl as one mass of black, mattery, ichorous, and mal-odorous scabs and incrustations, neither eyes nor any other features to be distinguished, and, in fact, the entire individual, reduced to the state of a putrid carcase, before the lenient hand of death put an end to such indescribable loathsomeness. This might compete with the pictures of blue cholera presented to the public every day, while all the minor grades of variola, with all the diarrhœas and other modifications of cholera were purposely kept in the back ground. Would this be fair? Certainly not. Numerous examples might be collected from the experience of this year, where fatal cases of cholera occurred in various parts of England, scarcely, if at all, to be distinguished from the Asiatic cholera. We shall quote one or two. A journeyman tailor was walking along Hanover Street in November last, and was taken so ill as to be carried to St. James's Workhouse, in Poland Street, with cholera. The surgeon, Mr. Braine, was so struck with the symptoms presented by this man that he ran for the Editor of this Journal, after he and Mr. Hillman had taken the proper steps of treatment. The man's face was pale and blue—his features shrunk—a dark circle round the eyes—skin cold—pulse imperceptible—stomach and bowels ejecting watery fluids—cramps so painful as to cause him to roar out—tongue and breath cold. Such was the condition of this man when received into the Workhouse; and though he had been in the warm-bath, and had brandy and laudanum exhibited before Dr. Johnson arrived, his state was little altered, except that the pulse had become perceptible, and the skin was warmed by the bath. The magistrates of Marlborough Street were eager to have a decided opinion on this case; but neither Dr. Johnson nor the other medical attendants could say that it was not one of the true Asiatic cholera. In fact, it presented every feature of the disease as it now prevails in Sunderland. In two hours the medical attendants had another consultation, and now things wore a more favourable aspect. The pulse was more developed, the skin warmer, as well as the breath—he had only been three times sick and purged in the two hours—the spasms were mitigated—the man was recovering. The patient's wife, meantime, had arrived, and informed us that her husband had eaten a quantity of pickled pork and hard cheese for supper the preceding night—and, moreover, that he had had a similar attack, though much slighter in degree, ten days previously. We were now in a condition to certify, for the information of the Secretary of State, that Hollis the tailor laboured under cholera certainly, but not Asiatic or imported cholera. Next day the tailor was able to cut out a waistcoat for his wife to sew up. Now this man, in all human probability, would have died, had not the most prompt means of relief been obtained—and in that case, who could have distinguished the disease from Indian cholera? Who, we ask, could have discriminated the case when the man was brought to the Workhouse? We, who have seen the Indian cholera, could not.

We will state another case still more remarkable. A medical practitioner residing in Piccadilly, or at least in partnership with a surgeon there, went, in August last, to hear Paganini at the Opera-house. He afterwards went to bed in his usual health. Next morning, when his wife awoke, she was petrified on looking at her husband, who appeared more like a corpse than a living being! He was sleeping calmly, though so shrunk and altered (he was a young man) that she scarcely knew his features. She sat by him for nearly an hour, not daring to wake him. The instant he awoke, he cried out for the wash-hand basin. Vomiting and purging commenced—all the phenomena of Indian cholera presented themselves (so at least we are informed by his partner) and he died that night.

Mr. Pretty, of Mabledon-place, related in the Westminster Med. Society a fatal case of cholera which occurred in his practice in the present Autumn, exhibiting all the symptoms of the Indian cholera. The patient survived the blue or cold stage, but died in the next or stage of re-action. We, ourselves, witnessed a case, so far back as July last, in a lady, who presented the features of the Indian cholera so exactly, that we put it down as one that would be almost certainly fatal. She recovered after being apparently in the jaws of death. From communications which we have received from various practitioners in town and country, we could multiply these examples to a very great amount; but the above authentic instances, coupled with the general disposition to similar complaints, which has prevailed for several months, will be sufficient to prove that the medical constitution of the present year has been eminently choleric, gastric, or intestinal, whichever term we may adopt. The case which we quoted at page 131 of this number, from Dr. Marshall, was acknowledged to be one of English cholera; but let the reader carefully re-peruse the symptoms, and he will find every one there that appertains to the Indian cholera—*even the total suppression of urine for three whole days before death!* Dr. Daun saw this case, and decided that it was *not* Indian cholera. Would he do so now? We ask Dr. Daun what symptom of the Asiatic cholera was wanting in Dr. Marshall's case?

But to return. Our readers will perceive by reference to the article on cholera, that, in Orenburg, Moscow, Warsaw, and other places, there was a prevailing disposition to stomach and bowel-complaints, previously to, and contemporaneously with the epidemic. The same disposition now unquestionably obtains in this country. Can this be accounted for on the supposition of a contagious principle communicated from individual to individual, and consequently beginning in one fixed point, and thence radiating, by means of men or goods, to all other points? We think this is a very narrow or confined view of the subject. Is it not more rational and accordant with experience, to attribute this as well as the other epidemics to some general cause, of which we are ignorant, while the emanations of the sick, in crowded, filthy, and unventilated apartments, endow the epidemic with a malignity and even an infectious character, not attendant on its origin, or essential to its nature? If it arose in Jessore, from causes beyond our ken, might it not, or rather did it not, arise from similar causes, in Calcutta, and hundreds of other places? We have the authority of Dr. Brown and others, that cholera prevailed in Sunderland and other places in the vicinity, to a very unusual extent, during the late Summer and Autumn. Can we much wonder that, in such places as Dr. Barry and others have described, where whole families were huddled together in one room, with little food and less cloathing, a malignancy and infection were superadded to the original epidemic? The same takes place in fever, in dysentery, in erysipelas, in scarlatina, and in many other diseases. Did not Dr. Barry declare, in the Westminster Medical Society, on the 10th of December, that the cholera of Sunderland resembled much more a malignant fever than the common cholera, as described by ancient and modern authors?

There can be little doubt, indeed, that in the crowded, populous, and wretched habitations of man, both in Asia and Europe, cholera, as well as all other diseases, under an epidemic influence, must and will assume new and additionally fatal symptoms; so much so, indeed, that we do not wonder at medical men declaring their belief that the cholera, for example, is a new disease, *sui generis*, never before witnessed on this earth. There is nothing *new* in this creed. Chisholm christened a fever on the coast of Africa by a new name (Bulam) and maintained that it was *sui generis*. He maintained that it traversed the Atlantic, and spread in our West Indian Islands, as well as along the shores of North America. This doctrine was maintained by hundreds of proselytes at that time; but is now laid in the tomb of the Capulets!

It has been argued in our medical societies, and particularly by Dr. Copland, that *the* cholera morbus of 1817, was never before witnessed in India. Yet all the essential features of the disease are described by Paisley, Curtis, and others—while we have the authority of Sonnerat that, in one *epidemic* visitation of cholera *sixty thousand* men died of it on the Coromandel coast in one year! Curtis, Paisley, and Johnson have described the "coldness of the skin"—"the shrinking of the features"—"the watery purgings"—"the cessation of the pulse"—"the sinking of the eyes"—"the cold and *shrivelled* fingers and toes" — "the laborious breathing"—"the *livor of the countenance and extremities*"—"the spasms" —and the fatal termination of the disease in "four or six hours." What more would Dr. Copland wish?

It is said by some writers that a peculiar smell issues from the bodies of choleric patients, and which proves it to be a disease *sui generis*. Let us hear what Mr. Annesley says on this point—an authority which Dr. Copland will be the last to question.

"Upon opening the abdomen, a peculiar offensive odour *was sometimes observed*, particularly in *those who died suddenly*."

Now who has not observed a peculiar offensive odour on opening the abdomen of people in this country who have died of other diseases than cholera? We have the authority of Dr. Hamett, who witnessed the disease on a large scale at Dantzic and other places recently, that this peculiar odour is *rarely* perceptible.

It has been argued also that the secretion of bile distinguishes the Asiatic from the cholera of this country. In the Asiatic disease, say they, no bile is secreted, or at least appears in the vomitings and purgings; while, in the cholera of this country, deluges of bile come forth. Nothing can be less correct than the latter assertion. Those who make it, have not observed accurately the train of phenomena, *from the beginning*, in severe—indeed, we might say, in the *common cholera* of this country. The advocates of this doctrine laud Sydenham as a careful observer of Nature—but they are forced to acknowledge that he never even mentions *bile* as an ingredient in the ejected matters from the stomach or bowels in cholera. The real state of the case is this. When a man is seized with cholera sporadica in England, the *contents* of the stomach and bowels are first ejected—then watery fluids, or whatever he has taken in—and *after some hours of straining*, bile occasionally or frequently appears in the discharges upwards and downwards. What is there here more than we see in a healthy person who has taken an emetic? We long ago shewed that bile was not the *cause* of cholera in this country (though generally considered so)—that it was usually absent in the cholera of India — and that, though a secondary or ternary link in the chain of phenomena, it was a favourable symptom in all countries and cases. If we look to one of the oldest and also to one of the latest writers on cholera —Aretæus and Bateman, we shall find the above statement confirmed. "In *primis* (says Aretæus) quæ evomuntur, *aquæ* similia sunt; quæ anus effundit, stercorea, liquida, tetri odoris sentiuntur. Siquidem longa cruditas et malum excitavit, quo si per clysterem eluantur, *primo pituitosa,* MOX *biliosa* feruntur." *De Cholera.*

Dr. Bateman, in Rees' Cyclopædia, remarks :—" The bowels are seized with griping pains, and the stools, which are at first *thin and watery,* as in common diarrhœa, are passed frequently. The stomach is seized with sickness, discharges its contents, and rejects what is swallowed. *In the course of a few hours,* the matter vomited, as well as that which is discharged by stool, appears to be pure bile."

But why need we consult authors when Nature is before us. Let any one who reads these lines accurately observe a case of cholera, *from the very beginning,* and he will find Dr. Bateman's description correct. But medical men seldom see a case till after several links of the chain are broken, and hence they have too often described from the middle of the case, instead of from the beginning. Dr. Barry very properly stated in the Westminster Society, that the absence or presence of bile was not essential in the blue cholera. He often saw healthy bile passed —and the gall-bladder was generally turgid with bile; but the gall-ducts were almost always impervious. Our readers will see how exactly this tallies with the TRINIDAD FEVER, as described by Dr. Hacket, in our EXTRA-LIMITES.

The relative mortality of this disease in India, on the Continent, and in England, deserves a remark. In India the average was 1 in 8. In St. Petersburg, Warsaw, and Hamburgh, it was more than one in two—while in England, up to the 14th December, it was scarcely 1 in 3. How are we to account for this? In India, we may be sure that the ratio of mortality was chiefly drawn from our military establishments, native and European—for it is not likely that the mortality of cholera could be ascertained, with any degree of accuracy, among the native population.—Now the military class of people, both Asiatic and English, were those who would be much more likely to resist the choleric poison, from their habits, age, and other circumstances, than the lower grades of society in any country. To these circumstances may be added the absence of all fear of contagion, and the prompt medical measures, voluntary assistance, &c. which were afforded. In addition to all these, let it be remembered that the first and most dangerous stage of cholera resembles, in all important points, the cold stage of a pernicious intermittent. A high range of temperature must surely be, in many respects, favourable to the restoration of the circulation, on which the safety of the patient depends. This, therefore, may be one cause why the ratio of mortality was, upon the whole, less in India than in Europe. In the *former,* too, the ventilation and cleanliness were infinitely greater than in the latter. Hence there was less of the typhoid fever that succeeds reaction—hence there was less infection in the disease. In Europe, and especially in Winter time, among the poor, the means of promoting reaction are infinitely more difficult than in a tropical climate—while, in crowded and filthy places, as in Sunderland, Newcastle, and Shields, the chances of infected atmospheres are, at least quadrupled. Notwithstanding this, the mortality has hitherto been much less than on the Continent. And we have reason to hope that, although the disease may be more extensive in Summer than in Winter here, the ratio of mortality will be less.

We shall now sum up the *conclusions* to which we have come, respecting the nature and treatment of the epidemic cholera *morbus,* or perhaps more properly speaking, the CHOLERIC-FEVER, in the form of a series of propositions submitted to the Westminster Medical Society on the 26th November last, by the Editor of this Journal.

PROPOSITIONS.

" I. That in *epidemic* cholera, as in most other epidemics, a poison or sedative principle, whether emanating from the earth, from animal or vegetable bodies on the earth, or engendered in the air, strikes a predisposed individual, and, after an uncertain period of incubation, produces a train of phenomena, forming the subject of subsequent propositions. In *sporadic* cholera, the general or diffusive cause is absent; but when the common exciting causes are strong, and the subject highly predisposed, severe or fatal cases will occur, where the *symptoms* cannot be distinguished from those of the epidemic cholera.

II. The effects of the choleric poison exhibit a great analogy to those produced by the virulent contagion of typhus, and the concentrated miasmal exhalations that give rise to malignant fevers, remittent and intermittent; such as have been seen in Batavia and other highly malarious places.

III. This poison shows its effects according to the evidence of our senses, *first*, on the nervous system, as evinced by the prostration of strength,—by the affection of the head,—by the arrest of the secretions (dependent on nervous energy), and, in fact, by a depression of the whole of the sensorial functions, as well as those of the organic life.

IV. The *secondary* effects of the choleric poison are shown in the vascular system. The heart acts feebly,—the circulation recedes from the surface, and the blood accumulates in the vessels of the internal organs; decarbonization and calorification cease, or are greatly diminished; the temperature of the body falls to that of surrounding inanimate substances; paleness is changed to blueness; and the influence of the ganglionic system of nerves seems to be nearly suspended, if not annihilated.

V. It is at this period that nature appears to make violent, but too often unsuccessful, efforts, to restore the broken balance of the circulation and to re-establish the secretions, by sickness and purging; the ejected fluids being exudations rather than secretions.

VI. If nature does not succeed by the above-mentioned efforts to restore circulation, secretion, and consequently oxygenation and calorification, these efforts themselves prove auxiliaries to the choleric poison in destroying life.

VII. We are not, in our present state of knowledge, certain whether the spasms be merely the effects of the poison on the nervous system, or an effort of nature to resist it; but they, like the sickness and purging, tend ultimately to exhaust the powers of life.

VIII. If nature, (by which I mean the constitution), whether with or without aid, be able to resist the first or depressive shock of the poison, and institute a reaction in the system, that reaction, in a great majority of cases, becomes a fever, exhibiting a new train of phenomena, and demanding a different mode of treatment. If this view be correct, it would lead to the inference that the choleric symptoms constitute the first or cold stage of a choleric fever.

IX. If reaction, with restoration of circulation, secretion, and oxygenation, do not take place, the patient dies in a state of asphyxia, the intellectual powers often remaining but little impaired, till the last glimmer of the lamp of life is extinguished. This has been often witnessed in concentrated miasmal fevers, both within and without the tropics.

X. Pathology.—All the changes which present themselves in the dead body are, in my opinion, *effects*, not causes, of the disease; with the exception of the congestion of black blood in the internal organs, which is almost the only phenomenon observable when cholera terminates fatally in a few hours. The traces of inflammation in various organs after death, indicate the causes or effects of the reactive fever, rather than of the cholera which precedes that fever.

XI. Treatment.—As we have no means of expelling or neutralising the poison, we can only endeavour to counteract its effects, and to assist nature in her remedial movements.

XII. The primary or essential indication is to restore the equilibrium of the circulation. That equilibrium effected, a restoration of secretion, calorification, and oxygenation, follows.

XIII. The balance of the circulation is to be restored partly by internal, partly by external means; but always by several means simultaneously employed at the very earliest period of the disease.

XIV. Venesection may appear a desperate remedy, but we have a desperate disease to combat. I proposed this measure many years before the epidemic broke out, and it has been adopted to a very considerable extent, both in Asia and Europe. I proposed, and would still propose, venesection with a two-fold view: first, to relieve the heart and internal organs from a portion of that deluge of black blood in which they may be said to be drowning;—secondly, to turn, as it were, the tide of the circulation from the centre to the surface of the body. This measure I would chiefly confine to the young, the robust, and the previously healthy, and in them contemporaneously with, or subsequently to, the measure which forms my next proposition.

XV. The first internal remedy which I propose, both in aid and in imitation of nature, is a stimulant emetic, as infusion of mustard seed, or what perhaps would be better, the sulphate of zinc. I propose this from a conviction founded on observation, that of all the means which nature or art can bring into operation, the act of *full vomiting* is the most powerful in driving the blood from the trunks to the capillaries—from the internal organs to the periphery of the body. It is also the most universal excitant of secretion in every glandular structure of the living machine. Nausea and retching are quite different in their effects from the operation of full vomiting. Nausea and retching depress the power of the heart and nervous system, and prevent the blood from flowing to the surface; full vomiting impels the circulation with such force into the superficial vessels, that it is extremely difficult to stop the flow of blood from the orifice of a vein during vomiting. I have seen the blood come from a vein under such circumstances, with all the characters, or at least the appearance, of arterial blood. This proposition is well exemplified by sea-sickness, of which I have had painful personal experience. An unfortunate landsman bears a close resemblance, during the first storm at sea, to a man with cholera. He staggers about the deck, or clings to the railings of the lee-gangway, striving to keep down the rebellious heavings of his own stomach. But all wont do; up it comes; and during the first vomitings I have seen the blood gush from mouth, nose, and even the eyes of the sea-sick sufferer. From being actually blue with nausea, his face becomes red with vomiting. But the cause of the sickness still continuing, he ultimately becomes pale and exhausted; he is like a man who takes a fresh dose of tartar emetic after each paroxysm of vomiting. The wonder is that he does not die; some *have* died. It is curious that Cœlius Aurelianus, who gives the best ancient account of cholera morbus, places sea-voyaging among the causes of that disease.

It is but justice to state that Mr. Boyle proposed and practised *full vomiting* in the epidemic cholera ten years ago; and I have the very best authority for affirming that this practice, when it was pursued on the Continent, was eminently beneficial.

XVI. As soon as vomiting has produced its salutary effect on the circulation, or has failed to produce that effect, after a fair trial, I would propose diffusible stimuli with calomel and opiates, but not in immoderate doses. Brandy and laudanum, as the most readily procured, and the least likely to be loathed, are perhaps the best. But the choice of stimulants must be left to the practitioner; and the danger of inducing subsequent inflammation should be carefully borne in mind. Calomel alone would probably be the best medicine after the emetic.

XVII. The remedies above-mentioned, in moderate but efficient doses, seem to impart vigour to the heart and nervous system, through the medium of the stomach, while they mitigate the spasms, and restrain the useless or injurious exudation of fluids from the intestinal canal, the mercury changing that exudation into secretion.

N.B. Would not the inhalation of oxygen gas be beneficial?

XVIII. The external remedies are three—heat, friction, and counter-irritation. These three means should be employed, not only simultaneously with respect to each other, but contemporaneously with the internal remedies. They should also be so employed, that the patient may not be required to throw a single voluntary muscle into action. Every muscular movement, even that of sitting up in bed, is prejudicial, or absolutely dangerous, during the exhausting orgasm of cholera. An apparatus permitting the application of heat, friction, and counter-irritation, without the necessity for any muscular exertion on the part of the patient, will be shown to the Society.

Query—As the cramps and spasms are chiefly confined to the extremities, and as the exhausting pain of these cramps is in proportion to the contraction and swelling of the affected muscles, would not firm compression by a bandage mitigate the spasms? Patients in cholera often cry out for the most violent extension and pressure of the cramped muscles.

XIX. Prophylaxis. — Conceiving myself, as well as every member of this Society, bound to abstain from all discussion of the question of contagion, I shall condense the subject of prevention into four words,—temperance, cleanliness, ventilation, and fearlessness:—in fine, the pursuance of all those means which tend to preserve general health, and the avoiding of all those causes which predispose to the common, or indigenous diseases of our own climate."

In respect to the cause of the epidemic, we have no reason to alter the conclusions to which we have come on former occasions—namely, that the disease originates in causes of which we are ignorant—and over which we have no control—and that, in crowded, filthy, and ill-ventilated places, it takes on an infectious character, tending still farther to propagate, and heighten the danger of, the disease. This is the doctrine which we have always maintained, from an unbiassed consideration of facts. We shall be ready to alter our opinion, whenever our experience and reflection shall convince us that we are wrong. The disease has now come within the range of observation; and English practitioners will not have to ground their conclusions on reports from the banks of the Ganges, the Ural, the Wolga, the Euphrates, or the Nile, *only*—they will probably have ocular demonstration—and to that ordeal we cheerfully submit.

Observations on the Nature and Treatment of Cholera, drawn up by Drs. Russell and Barry, 13th December, 1831.

"Of the two great classes of functions performed by the organs of which man is composed, one only is attacked in this disease. The operations of the senses and of the intelligence are either left untouched, or are affected but in a secondary manner.

Those functions, on the contrary, by which existence as a living being is preserved,—those complicated powers, by means of which we are for ever appropriating and converting into a part of ourselves portions of the matter around us,—are all and at once deranged by the attack of this terrible malady. Nutrition is annihilated; respiration becomes difficult, irregular, and inefficient; the involuntary muscles no longer perform their task; the voluntary are drawn into contractions by other powers than the will; the blood ceases to circulate; its physical properties are altered; its serous portion is suddenly thrown out upon the intestinal mucous surface of the body; the secretions are all arrested; and animal heat is no longer produced.

Under such rapidly destructive, and almost universal derangement of function, the most energetic efforts should be directed to reproduce what the disease has rendered nature unable to keep up, viz.

1st. Fluidity, heat, and motion in the blood.

2dly. Regulated action in the voluntary and involuntary muscles.

Lastly, but above every other consideration, renewed energy in the nervous centre, the source of all vitality and function.

No remedy at all approaching to the nature of a specific has been as yet discovered for this disease. In fact, no one mode of cure can be usefully employed under all the circumstances of any disease. The grades of intensity and the grouping of the symptoms with which spasmodic cholera makes its attacks, vary with the conditions of the subject; its treatment, therefore, must vary with these grades and conditions.

The leading preliminary symptoms generally are, either diarrhœa, spasms, apoplectic vertigo with nausea, imperfect vomiting, or various combinations of these symptoms.

When the diarrhœa affords time for distinct treatment, it ought to be arrested at once by the most prompt and efficient measures;—by opium in moderate doses; astringents; local bleeding by leeches, if the subject be plethoric; by cordials and sulphate of quinine, if there be cold sweats; by confining the patient strictly to bed, and keeping up heat; by diet; by emetics.

Should spasms be the first and leading symptom, subnitrate of bismuth, cupping along the course of the spine, cordial, and antispasmodic medicines, opium, frictions, and dry warmth are indicated.

But when the patient is suddenly seized with vertigo, nausea, coldness, loss of pulse, blueness of the skin, shrinking of the features and extremities, with more or less watery discharges and cramps; constituting an aggravated case of the worst type; whether this state shall have come on without warning, or shall have supervened upon either or both of the preliminary sets of symptoms already mentioned, time must not be wasted upon inert measures. Such a patient will inevitably perish, and within a few hours, if the paralysed vital functions be not quickly restored.

Let him then be immediately placed between warm blankets; and should no medical person be at hand, let two table-spoons full of common kitchen salt, dissolved in six ounces of warm water, be given immediately, and at once if he be an adult. Let dry and steady heat be applied along the course of the spine, and to the pit of the stomach (if no other means be at hand) by a succession of heated plates or platters. Let the upper and lower extremities be surrounded with bags of heated bran, corn, ashes, or sand, and assiduously rubbed with a warm hand, and a little oil or grease to protect the skin. Energetic, complete vomiting will probably be produced by the salt; and perhaps bilious purging, with tenesmus.

Should a medical man be on the spot, a moderate bleeding, if it can be obtained, would be desirable, previously to or immediately after the administration of the salt, or of any other emetic which may be preferred.

The extensively deranged action of those organs whose nerves are chiefly derived from, or connected with the spinal marrow, the anatomical characters found about that great source of vitality after death, in many cases of this disease, together with the success stated by Dr. Lang, chief physician at Cronstadt, to have attended the practice mentioned below, founded upon these views, in 12 out of 14 aggravated cases, fully justify the following recommendation.

In cases such as those just described let the actual cautery be freely applied to one or two or more places on either side of the spine, as if for the purpose of forming good sized issues. Should the heated iron have produced any excitement of the nervous power, and the salt emetic have caused any portion of the bile to flow through its proper duct, a great step will have been accomplished towards recovery from the stage of collapse. Cordials and opiates judiciously administered; sinapisms and other external stimulants; mercurials with mild aromatic aperients, which the intelligence and activity of British medical practitioners will not fail to adapt to the actual circumstances of each case, will conduct the patient safely to the stage of re-action.

The organs during the collapse of this disease, probably owing to deficient vitality, often give no indication of having been acted upon by repeated doses of certain powerful medicines, which under other circumstances would have produced the most decided effects. It is therefore suggested that this temporary insensibility of the system should not inculcate the administration of such quantities as could, by accumulation, when the organs begin to recover their vitality, give rise to unfavourable results.

Thirst being a most distressing symptom of this disease, the quality and temperature of the drink should perhaps be left to the choice of the patient; but the quantity taken at a time should not exceed four ounces, and should be acidulated with nitrous acid, if the patient will bear it.

Should the disease prove extensively and rapidly epidemic in a large community, it would be prudent to establish stations at convenient distances from each other, where medical assistance and medicines might be procured without the risk of disappointment or delay. The details of these arrangements are left to the wisdom of local boards of health.

As to the symptoms of the consecutive stage of feverish reaction in cholera, they differ but little, if at all, from those of ordinary typhus, except, perhaps, in the greater rapidity with which they but too often run to a fatal termination; and as this kind of fever is treated in no part of the world with more success than in England, the entire management of this stage of the disease is left to the zeal and science of the profession at large.

Attentive nursing, and assiduous, well-directed rubbing, are of the utmost importance; a strictly horizontal position, however, must be maintained until the heart shall have, partly at least, recovered its action. An erect or even semi-erect position, during the collapse, has been often observed to produce instant death. Warm baths, therefore, for this and other reasons, are worse than useless; evaporating fluids, and indeed all moisture applied to the skin, seem to be contra-indicated for obvious reasons. Hot air baths, so contrived as to be applicable in a recumbent posture, and admitting access to the patient for the purpose of friction, may be of use.

I have the honour to be, Sir,
Your most obedient Servant,
E. STEWART, Chaiman."

Our readers will readily perceive that the above document presents exactly the same views, as to the nature and treatment of cholera, which Dr. Johnson's propositions contain, as laid before the Westminster Medical Society on the 26th November last. The preponderance of disease or rather oppression in the ganglionic system of nerves and the functions thereby supported—the comparative immunity of the sensorial or intellectual functions—the arrest of secretion, calorification, secretion, and oxygenation—the intestinal *exudations*—in short, every pathological phenomenon contained in Dr. Johnson's propositions, is embodied, and in nearly the same terms, in the code of the Central Board of Health. We challenge any one to point out a single iota of discrepancy. This is no trifling source of gratification. But there are some parts of the document which, we apprehend, are not very guardedly composed. Surely Drs. Russell and Barry were hasty in declaring that, in cholera, "the blood ceases to circulate." The instant that the blood *ceases* to circulate, syncope or death ensues. Some degree of circulation *must* continue, so long as the heart continues to act—when the heart ceases to act, circulation and all the phenomena of life cease also. A man in syncope is dead while syncope lasts. The expression, therefore, of the Board is too strong—and is not correct.

Again, the Board avers that the sensorial or intellectual functions are not at all, or only secondarily affected in cholera. How does this assertion quadrate with the information that "the leading premonitory symptoms" include "apoplectic vertigo." But appealing from words to things, we maintain that the impression of the cause of cholera, whatever it may be, is manifested on the *whole* of the nervous system—cerebral as well as ganglionic—though the *latter* is most affected. The prostration of strength, which invariably shews itself at the very onset of the disease, is peculiarly the result of depression of the cerebral system. The spirits also are depressed, however clear the intellectual faculties may remain in the course of the disease. Besides, how is it that the spinal marrow, which is merely a prolongation of the brain, and as much unconnected with the ganglionic system as the brain itself,—how is it, we say, that this organ is pointed out as a main seat of the disease, and the actual cautery recommended in its vicinity? Surely the spinal nerves are more connected with voluntary motion than the ganglionic nerves. We have the authority of Mr. Searle and the French physicians, that the spinal marrow shews no particular signs of disease—and we venture to predict that dissection, in this country, will shew the hypothesis, for such it is, to be totally untenable. Indeed the Board itself proves this, by considering the ganglionic system of nerves to be chiefly and *primarily* affected. So much for the pathology. In respect to therapeutics, the Board has prescribed precisely the same means which Dr. Johnson pointed out. External heat, friction, and counter-irritation, with rigid adherence to the horizontal position. As to internal remedies, Dr. Johnson's proposals are almost all adopted. Emetics—venesection —and afterwards cordials, mercury, &c. are recommended.

Upon the whole, and with the few animadversions which we have made on certain pathological points, we have great reason to be gratified that none of our essential therapeutical recommendations have been contra-indicated by the BOARD OF HEALTH.

PICTURE OF CHOLERA IN SUNDERLAND.

WE have alluded to the impropriety of drawing pictures from the extreme cases of any disease, viz. small-pox, and holding them up *in terrorem*, leaving in the back ground all the minor but more numerous shades of a malady. This has been done with cholera by too many of our medical portrait painters. The following curious picture of cholera in Sunderland has lately been published by Dr. Tinn of Newcastle. We must somewhat abbreviate the document.

Anxious to view this terrible malady, Dr. Tinn repaired to the cholera hospital in Sunderland, where five cases were said to be in *the second stage* of the disease.

The *first* patient was Edward Downs, aged 85, who had been ill for some time, but became worse on Friday night, 9th December. This patient, from the symptoms which he detailed, appeared to have had some inflammation of the mucous membrane of the bowels. "His abdomen was soft and natural—some pain there on pressure — occasional pains in his legs—tongue dry but clean —thirst—pulse full and natural—skin warm and natural." *This was the second stage of cholera.* Dr. T. avers that "he never had any symptom of the first stage"!

*Second Case.* Mary Wildon, aged 70, says that she has occasional pain in her head—no pain in any other part—pulse natural—tongue clean and moist—skin natural. "In my opinion there was nothing the matter with this woman, nor did she appear to have had any disease whatever." *Still she was in the second stage of cholera.*

*Third Case.* Eleanor Appleby, aged 63, an idiot. She complains of pain in her back—no appetite—thirst—tongue red in the middle, but furred at the edges—pulse small and quick—pain in her side—bowels hard and contracted. "She never has had any symptom of cholera morbus." But she is now in the second stage.

*Fourth Case.* Susan Roach, aged 40, says she has been out of health for some time. Tongue is moist and clean—skin warm and natural—pulse small and rather quick—pain in her left side—has been sick this morning — no purging. Second stage of cholera.

*Case five.* Eliz. Snipe, aged 20. Has been a servant in the hospital since its commencement—is stout and healthy-looking—has been rather poorly since Monday last—complains of pain in her head—pulse rather quick—skin warm and moist—tongue clean, &c. Dr. T. observes that—"there was not a single symptom or appearance that could induce any one to believe that she was in the second stage of malignant cholera, and I think it is clear that she never had the primary stage."

Dr. T. now went in pursuit of a case in the first stage, and applied to Mr. Embleton, who attends the poor, and reported eight cases on Sunday, Dec. 11th, 1831. Two of these cases Dr. T. had an opportunity of seeing on the same day, in company with another medical gentleman. One of these was a fish-woman, aged 70, "who lived in a most miserable situation." She had been in the habit of taking "something comfortable," whenever she could get it. She had sickness—dry and furred tongue — loose fetid evacuations — no pain in the stomach or bowels — no spasms—no cold skin—no suppression of urine—no blueness—no prostration of strength—no shrinking of the fingers or toes—"in short, no symptom of cholera." "This was one of the cases reported under the term malignant cholera." The other case was a man of the name of Nicholson, residing in Sailor's Alley, "whom we found with no symptom whatever of cholera, either of the first or second stage."

Dr. T. winds up in the following words: "It is almost impossible to repress our indignation that such common and every-day occurrences should have excited so much unnecessary alarm." "Mr. Embleton attends the poor of Sunderland; it is no wonder, therefore, that he is called to so many old and poor people who are suffering at this season of the year from disease. If I may be allowed to form an opinion from what I could observe yesterday (Sunday) the malignant cholera morbus is not likely to subside so long as there is disease or death in Sunderland."

We have not the smallest doubt that during the panic, almost every disease, and almost every death will be laid to the account of cholera morbus!! But a time will come when men will have leisure to portray the epidemic in all its shades and gradations, without exaggerated, or at least exclusive representations of its intensest forms. We have much need of a fair and unbiassed picture of the EPIDEMIC CHOLERIC FEVER, in the North of England!

The following extract from the TIMES reporter, (Dec. 15th), an ultra-contagionist, will afford some clue to the "picture of cholera" above.

"The proportion of *mild cases* that have occurred in Sunderland is, *beyond any ordinary measure of comparison*, greater than those recorded during the inroads of the cholera in India."* "In Sunderland, the mild cases have been met with during the whole period of the invasion, and they have often appeared in the same family of persons, or in the same locality, with those of the more dangerous character. In this way, I have observed that one member of a family may have the disease commencing slowly in the shape of a watery purging—another member may have, at the first onset, the mild symptoms of cholera fully developed; while a third will have the attack in the most rapid and violent type."

This speaks pretty plainly as to the number of cases in Sunderland and Newcastle. It shews clearly that the epidemic is a gastro-intestinal disease, assuming all forms and grades, from the simplest diarrhœa up to asphyxial fever, according to the predisposition of the individual, the insalubrity of the locality, and the strength or dilution of the epidemic cause.

---

*How is Cholera propagated?* By an American physician. This little pamphlet, published by Mr. Millar, of Henrietta-street, Covent Garden, contains many sensible remarks. We shall endeavour to find space for a few short extracts.

"There are so many evidences of the causes of fatal fevers originating under certain conditions of the atmosphere, in all parts of our globe, and disseminated through the air, sometimes slowly, and sometimes rapidly, over vast regions,—the disease appearing in the same type at different places, with greater or less mortality,—that the fact may now be considered as well established. The medical history of England abounds with interesting matter upon this subject. That the Plague, Yellow Fever, and perhaps the Asiatic Cholera, thus originate and are disseminated, is very probable; for these diseases, if they were communicable, like Small Pox, and capable of being multiplied like it, through the morbific secretions of the body, would long since have visited the four quarters of our earth with the most awful destruction of the human family. That infectious diseases, in their progress, obey very different laws, is, however, evident from the well-recorded testimony we possess of their manner of appearance and disappearance. They are all the endemics of certain latitudes; but sometimes change their characters, and become epidemic, and very mortal in their course."

The author thinks that, in respect to yellow fever, vessels lading at the wharfs of cities, where the disease prevailed, have taken it with them to distant climes, without infecting the crews, and then introduced it with the discharge of their cargoes. We apprehend that few people beyond the At-

* It is to be remembered that the mortality in India was infinitely less than in Europe.—Ed.

lantic entertain this opinion at present. The following passage contains more important truths.

"The first cases, in all epidemics of this description, are usually the most fatal; and where the infected air has been pent up in narrow and confined streets, the disease has often been observed to spread in a direction corresponding with the currents of air. A knowledge of this fact has led to the immediate removal of the citizens, and the boarding up of those streets in which the pestilential air is known to exist. A great portion of the population of the city of Baltimore was thus saved in the year 1819, when this disease appeared in that city in a mortal form."

"How the exhalations so noxious and fatal to human life are generated, it is best at once to say, we are perfectly ignorant; *causa latet, vis est notissima,* is perfectly applicable here; and, without entering into any theory or speculation upon this interesting subject, my whole aim is to direct the attention of the medical profession, at the present important crisis, to a strict observation of the manner in which the Asiatic Cholera is disseminated: for upon this correct knowledge must depend all preventive means and the safety of the population. It may be a disease communicable from body to body, and obeying, like Small Pox, all the laws of a contagious disease; but it has occurred to the writer, from what he has read upon the subject, that Cholera, like Yellow Fever, has in truth no common attribute of a disease strictly contagious, and that we must look for some other solution of the manner in which its germs, so fatal to life, are produced and sown."

"An early removal of the population from the sources of infection, and an immediate interdiction of the healthy from diseased parts of a town, are of the first importance in arresting many of our epidemic diseases; and, should the Asiatic Cholera prove thus communicable from one to another, will be the only efficacious means which can be resorted to, and by which we can arrest its course, or prevent its communication through the foul air of vessels coming from infected ports. The contagious nature of this disease has been rather hastily inferred from the fact of its following, both in Asia and Europe, the great rivers and masses of population; for it accords with observations both of ancient and modern times, that many diseases, such even as are not ranked as contagious, are diffused over Continents in a direction contrary to the currents of air, and with a certainty which defies all human means to avert or arrest, and that these diseases are generally mild when extensively diffused, and become malignant when the cause is concentrated. Now, it is not difficult to conceive that a concentration of the cause of Cholera, producing its malignant form, should take place in camps or in the crowded or ill-ventilated portions of cities, generally inhabited by the poorer part of the population, and that in this concentrated form it should be carried along the courses of rivers in vessels, or over the highways of nations. What medical man is there who does not know the fatal effects of confining any quantity of diseased persons to narrow and ill-ventilated apartments? Yet, will this be called contagion? Small Pox produces its impression fully wherever taken, and multiplies itself. Not so with jail or hospital fever, which we all know to be infectious only at the source whence it emanates, or through clothes which have not been properly ventilated."

The author appropriately instances the epidemic influenzas of the United States. They originate in the Autumn, on the borders of the great lakes, and after passing over the Continent, in a direction parallel with the sea, "with a certainty the most extraordinary," disappear in the milder climate of the Gulph of Mexico; or are lost in the boundless forests beyond the Mississipi.

"The Influenza, in its course, rarely spares any of the population, but is seldom fatal, except in cases of old people. So regular is its march, that its appearance in one town is the certain signal of the next becoming afflicted with it, and sooner or later, according to the distance which separates them. The singular appearance, also, a few years ago, in the Antilles, of a fever with rheumatic symptoms, familiarly called the Dangue, which, after passing through the islands, visited the coast on the Gulph of Mexico, and extended itself from the sea to the mountains, and as far north as the Capes of the Chesapeake, is another instance. The universal diffusion of this disease was so remarkable, that few, or none of the population escaped in those regions through which it extended. It was communicated through the atmosphere, and appeared capable of being propagated only where the climate was regulated by a certain degree of temperature, for no restrictions whatever were imposed upon intercourse of the population from south to north. This disease was believed to be highly contagious, and in the West Indies, where it first appeared, was declared to be imported, as all bad things are, from Africa."

The obscurity which attends the propagation of epidemic diseases, and which has puzzled the faculty in all ages, ought to teach our controversialists a little moderation. It will probably be found that, in cholera, as in other epidemics, all known laws will be insufficient to explain the mode of propagation. Let us carefully observe facts, and wait the great teacher, Time, for unravelling the mysteries of the reigning epidemic.

*P.S.* 17*th Dec.* 1831. Dr. O'Shaughnessey, who is now in Sunderland, reporting on cholera, gives it as his opinion that the early cases of common cholera in Sunderland were the *same* disease as now prevailing, only in a milder form. Now these cases prevailed ever since July last, according to the testimony of the best physicians in Sunderland and Newcastle. This admission cuts off one head of the hydra—the *importation* from Hamburgh, for the disease did not then prevail there. Dr. O'Shaughnessey very appropriately compares the effects of the choleric poison with the effects of any other poison in small, moderate, and excessive doses. Does this quadrate with a specific poison generated in each individual, or with a poison diffused generally in the air—in other words, an epidemic influence? That an epidemic disease, often of a most malignant and fatal kind, prevails in Sunderland, Newcastle and vicinity, there can be no doubt. But that it softens away into all the shades between "blue cholera" and the most common stomach and bowel-complaints—all comprehended in, and reported as, the new disease, we have also no doubt.

We do not subscribe entirely to the Chinese philosophy, which looks upon every epidemic as a blessing from Heaven, to carry off the redundant population; neither do we believe, with Dr. George Gregory, that cholera was sent by Divine Providence, like small-pox, to scourge mankind, and to remain with man as a fatal legacy*—but we are among those Optimists, described in Rasselas, who believe that "every thing is for the best"—and we firmly believe that cholera, if it pursues its usual course, and selects its usual victims, will, in the end, do infinite good. It will strike the drunken, the debauched, and the profligate, in the *lower* classes of society—and if it invades the *upper* classes, the same description of people will be its victims. That a small proportion of innocent, virtuous, and temperate people will suffer, there can be no doubt; but the tares rather than the good grain will assuredly be swept away. Do the godly accuse us of cold and calculating philosophy? We refer them to Scripture. A sparrow cannot fall, without the permission of Providence—much less man, in thousands and tens of thousands.

But mankind, however religious, have a wonderful propensity to take physical means of guarding against disease. The cholera, which seems to visit intemperance and filth with inexorable cruelty, will have more effect in restraining or removing these *predispositions* than all the harangues from the pulpit—than all the precepts from the mouths or pens of physicians!

---

* Dr. Gregory, with great naiveté, offered his opinion, in the very same breath, in the Westminster Medical Society, that, if a wall (he did not state the height) could be erected round Great Britain, the same scourge, sent by Providence, would be kept out!!

